# Amino Acid Sequence Controls Enhanced Electron Transport in Heme-Binding Peptide Monolayers

**DOI:** 10.1021/acscentsci.4c01849

**Published:** 2025-04-02

**Authors:** Hao Yang, Xiaolin Liu, Moeen Meigooni, Li Zhang, Jitong Ren, Qian Chen, Mark Losego, Emad Tajkhorshid, Jeffrey S. Moore, Charles M. Schroeder

**Affiliations:** † Beckman Institute for Advanced Science and Technology, University of Illinois at Urbana—Champaign, Urbana, Illinois 61801, United States; ‡ Department of Materials Science and Engineering, University of Illinois at Urbana−Champaign, Urbana, Illinois 61801, United States; § Department of Chemistry, University of Illinois at Urbana−Champaign, Urbana, Illinois 61801, United States; ∥ Center for Biophysics and Quantitative Biology, University of Illinois Urbana−Champaign, Urbana, Illinois 61801, United States; ⊥ School of Materials Science and Engineering, Georgia Institute of Technology, Atlanta, Georgia 30332, United States; # Renewable Bioproducts Institute, Georgia Institute of Technology, Atlanta, Georgia 30332, United States; ¶ Department of Chemical and Biomolecular Engineering, University of Illinois Urbana−Champaign, Urbana, Illinois 61801, United States; ○ Chan Zuckerberg Biohub Chicago, Chicago, Illinois 60642, United States; △ Department of Biochemistry, University of Illinois at Urbana−Champaign, Urbana, Illinois 61801, United States

## Abstract

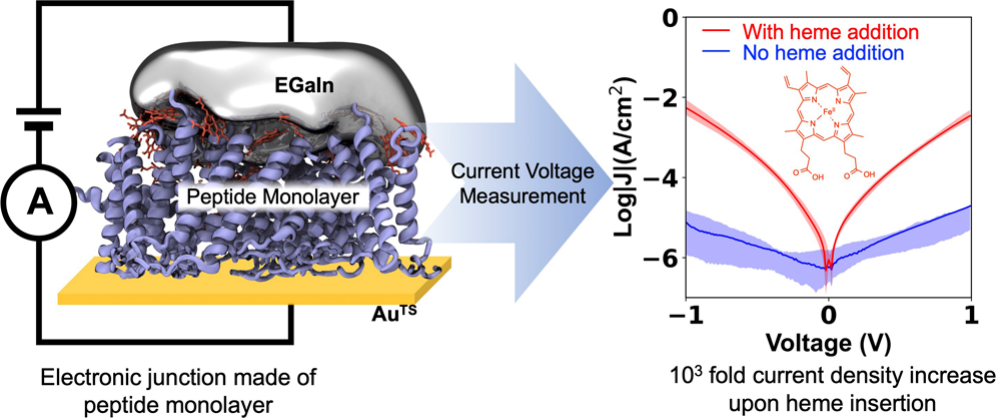

Metal-binding proteins have the exceptional ability to facilitate long-range electron transport in nature. Despite recent progress, the sequence-structure–function relationships governing electron transport in heme-binding peptides and protein assemblies are not yet fully understood. In this work, the electronic properties of a series of heme-binding peptides inspired by cytochrome *bc*_1_ are studied using a combination of molecular electronics experiments, molecular modeling, and simulation. Self-assembled monolayers (SAMs) are prepared using sequence-defined heme-binding peptides capable of forming helical secondary structures. Following monolayer formation, the structural properties and chemical composition of assembled peptides are determined using atomic force microscopy and X-ray photoelectron spectroscopy, and the electronic properties (current density–voltage response) are characterized using a soft contact liquid metal electrode method based on eutectic gallium–indium alloys (EGaIn). Our results show a substantial 1000-fold increase in current density across SAM junctions upon addition of heme compared to identical peptide sequences in the absence of heme, while maintaining a constant junction thickness. These findings show that amino acid composition and sequence directly control enhancements in electron transport in heme-binding peptides. Overall, this study demonstrates the potential of using sequence-defined synthetic peptides inspired by nature as functional bioelectronic materials.

## Abstract 2

Self-assembled peptide monolayers show a 1000-fold increase in electronic conductivity upon heme binding, demonstrating that electronic properties in bioelectronic materials can be tuned by controlling peptide sequence and composition.

## 1 Introduction

Electron transport is a fundamental biological process required for cellular respiration, photosynthesis, and energy transduction.^1^ Efficient and regulated electron transport is vital for cell survival and energy metabolism. In nature, electron transport is facilitated by complex electron transport chains, where electronically active proteins such as cytochromes provide the essential pathways for electron flow. In photosynthesis, the cytochrome *b*_6_*f* complex serves as an electronic link between photosystem II (PSII) and photosystem I (PSI), transferring electrons between these protein complexes. This process not only transfers electrons from PSII to PSI but also helps to establish a proton gradient across the thylakoid membrane using the potential energy generated during electron transport.^2^ Similarly, in the mitochondrial electron transport chain, the cytochrome *bc*_1_ complex performs a dual function by transferring electrons while simultaneously pumping protons across the mitochondrial membrane, thereby creating the proton motive force essential for ATP production.^3^

Long-range electron transport is efficiently carried out in biological systems using protein-based nanowires. Metal-reducing bacteria produce extracellular cytochrome-based molecular wires that are capable of transporting electrons over distances exceeding 10 μm. *Geobacter*, a common soil bacteria, has developed microbial protein nanowires for long-range extracellular respiration and electron exchange.^4^ Recent work has shown that these bacterial nanowires are composed of ordered assemblies of heme-containing proteins such as OmcZ, OmcS, and OmcE cytochromes.^5−8^ A defining feature of these electronically active proteins is the presence of heme, which is an iron-containing metalloporphyrin that plays a crucial role in a wide array of biological processes including electron transport, catalysis, and redox reactions.^9,10^ The structure–function relationships of these electronically active proteins have long fascinated scientists because they offer valuable insights into fundamental biological phenomena while also providing inspiration for the development of bioelectronic materials.^11−13^ The use of natural biomaterials in bioelectronics is particularly compelling due to their inherent biocompatibility and ability to integrate seamlessly with biological systems. For example, in cell-on-chip systems, direct cell growth on electrodes often presents difficulties with material compatibility.^14−16^ Electronically active biomaterials offer promising routes to overcome these challenges by providing conductive and biocompatible interfacial layers, enabling efficient signal transmission and improving device functionality. However, many biomaterials are inherently nonconductive, which limits their utility in bioelectronic applications.

Inspired by these efficient long-range electron transport systems in nature, a compelling question arises: how can we develop new functional bioelectronic materials by designing peptide sequences and integrating metal-binding cofactors such as hemes or metalloporphyrins? Peptides provide a robust structural scaffold^17,18^ to incorporate heme units, thereby achieving effective functional doping to control the electronic properties of these materials. Subtle differences in amino acid composition can lead to profound changes in the functional properties of proteins, suggesting that molecular engineering approaches provide a promising platform to develop new peptide-based bioelectronic materials.

The electronic properties of peptide-based bioelectronic materials can be characterized using self-assembled monolayers (SAMs) on electrode surfaces.^19−22^ SAMs allow for controlled peptide orientation on metal electrode surfaces, which enables detailed investigation of how amino acid sequence, composition, and presence of cofactors influence electronic properties at the molecular level.^23,24^ In addition, the ability to create ordered and oriented interfaces using peptide-based SAMs allows for studying fundamental interactions between biomolecules and electronic components at the nanoscale.^25,26^ Applications involving peptide-based SAMs extend beyond basic research to include the development of advanced surface modifications for nonfouling surfaces and cell-on-chip technologies.^27−30^ Overall, the versatility and precision of peptide-based SAMs hold strong potential to enable new discoveries and innovations in bioelectronics.

In this work, we study the electronic properties of a series of sequence-defined heme-binding peptides inspired by cytochrome *bc*_1_ that are capable of forming secondary structures and coiled-coil helical bundles.^31−34^ Peptide SAMs are prepared on gold electrodes, followed by structural and chemical composition analysis of assembled monolayers. A liquid metal soft contact electrode technique based on eutectic gallium–indium alloys (EGaIn) is used to characterize the electronic properties (current density–voltage) of peptide-based SAMs.^21,35^ Heme incorporation into peptide SAMs increases current density by 3 orders of magnitude without altering the junction thickness. Enhancements in current density are governed by heme loading, as well as the sequence and composition of the peptide monolayers. These findings demonstrate that cofactor-binding peptides can be engineered to create bioelectronic junctions with tailored electronic properties.

## 2 Results and Discussion

**Figure 1:**
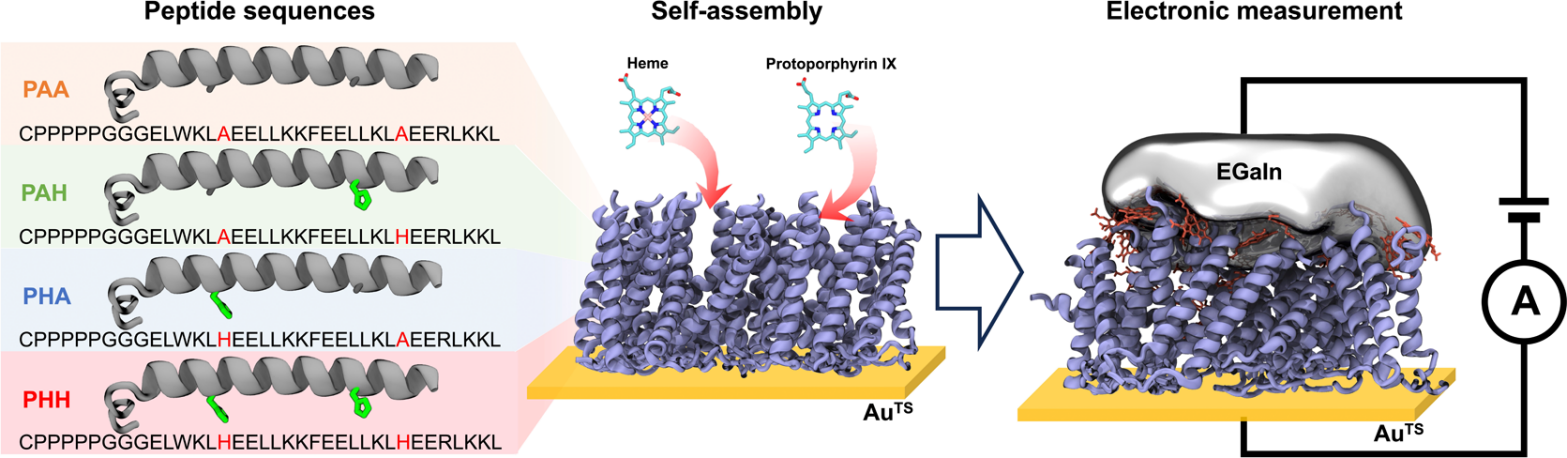
Schematics and primary amino acid sequences of peptides PAA, PAH, PHA, and PHH, illustrating their self-assembly on gold surfaces. Electronic properties were measured using a liquid metal soft contact method with eutectic gallium–indium (EGaIn).

We began by designing a series of α-helical peptides inspired by cytochrome *bc*_1_(31−33) as a model system to understand electron transport in heme-binding proteins (Figure 1). These sequences share similarities with cytochrome *bc*_1_ in the highly conserved residues of the wild-type sequence. These include histidine residues responsible for binding heme, phenylalanine residues that separate the heme-binding domains, and arginine residues that modulate the redox potential of the bound heme. These synthetic peptide sequences have been previously shown to specifically bind heme units by coordination to histidine residues in the core region of the α-helices.^32−34^ In this work, several additional modifications were made to the initial peptide sequence to enable SAM formation while preserving the core structure of the heme-binding helix. In particular, a cysteine residue was included at the N-terminus to facilitate binding to the gold electrode,^36^ and a short 5-residue polyproline sequence was added after the N-terminal cysteine to promote the formation of densely packed peptide SAMs by orienting the peptide chains in an upright position on the gold surface.^23^ The four peptide sequences are denoted as PHA, PAH, PHH, and PAA, referring to the polyproline sequence near the N-terminus and the identity of amino acids (histidine or alanine) at positions 15 and 29 in the modified peptide sequences used in this work. High-performance liquid chromatography and MALDI-TOF mass spectrometry were used to characterize the properties of synthesized peptides (Supporting Information, Figures S1–S2).

### 2.1 Peptides in Solution Exhibit Helical Structures and Heme Binding Ability

Prior to monolayer preparation and electronic characterization, we first examined peptide secondary structures and the ability to specifically bind heme in solution. Results from circular dichroism (CD) experiments (Supporting Information, Figure S3, Table S1) show that all four sequences exhibit two distinct negative peaks around 208 and 222 nm, which is consistent with helical peptide structures.^37^ We next investigated the heme-binding abilities of these peptides by performing UV–vis titration experiments. UV–vis spectra were determined upon incremental addition of hemin to an aqueous peptide solution (Supporting Information, Figure S4). As previously reported, heme coordination to peptides is achieved by adding hemin chloride directly to an aqueous solution containing peptides,^32−34^ and the coordination chemistry between heme and histidine induces a shift in the absorption spectrum of the heme unit.^32,33,37^ Our results show that as the hemin concentration increases, the absorbance peak around 413 nm increases while simultaneously undergoing a subtle hypsochromic (blue) shift.

To quantitatively characterize the UV–vis titration experiments, peak absorbance values at 413 nm are plotted as a function of the ratio of hemin to peptide concentration during the titration experiment (Supporting Information, Figure S5). The PAA sequence lacks a histidine for specific heme coordination, and the resulting absorbance values at 413 nm show a linear trend as a function of hemin concentration. In contrast, peptides PHA, PAH, and PHH, each having at least one histidine, give rise to pronounced changes in absorbance at 413 nm during titration as hemin concentration increases. An abrupt change in the slope of the absorption intensity marks the point of full coordination between heme and histidine. The ratio of hemin-to-peptide concentration ([hemin]:[peptide]) at which this transition occurs provides an estimate of the saturation binding ratio.^38,39^ Sequences PHA and PAH have only a single histidine per peptide chain, and the transitions in absorbance values during hemin titration are observed around [hemin]:[peptide]≈0.4. In contrast, sequence PHH possesses 2 histidines per chain, and the transition occurs at a larger hemin-to-peptide ratio [hemin]:[peptide] ≈ 0.9. Overall, these results are consistent with prior literature reporting the heme-binding properties of the parent peptides (without N-terminal cysteine and polyproline sequences).^33^

### 2.2 Peptides Form Uniform SAM Layers on Gold Surfaces

Following solution-phase characterization, we fabricated self-assembled monolayers (SAMs) of peptides on template-stripped gold surfaces (Au^TS^).^40,41^ Peptide SAMs were prepared by immersing Au^TS^ surfaces in a 0.1 mg/mL aqueous peptide solution (see Supporting Information, Section 7.2). In cases of heme incorporation (where noted), an aliquot of stock hemin solution was introduced into the peptide solution containing the Au^TS^ surface approximately 1 h after immersing the Au^TS^ surface in the peptide solution, thoroughly mixed, and incubated for 24 h.

**Figure 2:**
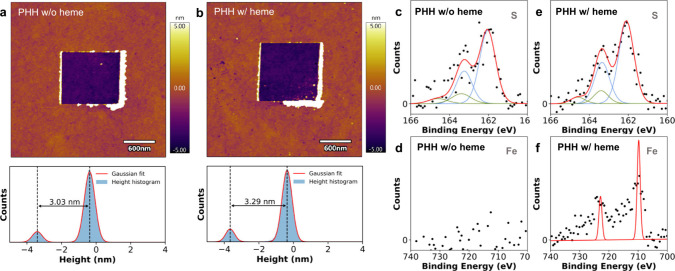
Structural and compositional characterization of peptide monolayers. AFM topography (top) and height histograms (bottom) of PHH monolayers (a) without heme and (b) with heme during SAM preparation. The thickness of the adsorbed peptide layer was measured by using an AFM tip to remove peptides from a central rectangular region. High-resolution XPS spectra for (c–d) S 2p and Fe 2p scans for PHH monolayers without heme, (e–f) S 2p and Fe 2p scans for PHH monolayers with heme. Raw data are plotted as the black points, and appropriate deconvolutions (green and blue) and overall fit (red) were plotted as the curves.

We used atomic force microscopy (AFM) to characterize peptide SAM morphology and thickness.^42^Figures 2**a,b** illustrate the morphology of PHH monolayers with and without heme incorporation. In general, AFM images reveal predominantly featureless surfaces, indicating that the peptides are effectively organized into uniform layers with no clustering or aggregation. To determine the thickness of the adsorbed layers, we used AFM tips to scratch a central region of the films, which effectively removes surface-bound molecules from a rectangular region on the gold surface (see Supporting Information, Section 4). A force of 100 nN was applied to the AFM tip during the scratching process. Our results show that this force is insufficient to cause significant damage to the underlying gold substrate (Supporting Information Figure S7). To quantify adsorbed layer thickness, section profiles are determined across the scratched and unscratched regions, and surface height distributions are plotted to reveal the height differences (Supporting Information, Figures S8–S11). Interestingly, our results show that monolayer thickness is nearly constant (around 3 nm) in the presence or absence of heme. The solid-phase monolayer thickness, compared to the solution-phase contour length estimate of peptide helices (≈5 nm based on the amino acid sequence),^43^ suggests that the peptides assemble into well-ordered monolayers with a small tilted angle normal to the electrode surface. In addition, attenuated total reflectance-Fourier transform infrared (ATR-FTIR) spectroscopy was used to study the peptide structure on gold surfaces. Prior work has shown that amide I IR absorbance strongly correlates with peptide secondary structure. Helical structures typically show strong absorbance within the range of 1648–1670 cm^–1^, whereas unordered or β-sheet structures are characterized by an amide I peak in the range of 1625–1648 cm^–1^.^44^ Our results reveal an absorbance peak at 1662 cm^–1^, indicating that these peptides retain their helical structures when assembled as a monolayer (Supporting Information, Figure S12). As discussed below, the structure of assembled monolayers plays a key role in electron transport, as monolayer thickness directly corresponds to the molecular junction distance for electronic measurements.

We next characterized molecular packing density in assembled monolayers, which is a key factor influencing electron transport in these systems.^45,46^ Whereas the monolayer thickness controls the distance over which electrons are transported in molecular junctions, molecular packing density determines the number of charge transport channels per area and plays a role in governing intermolecular interactions.^47,48^ It is desirable to maintain a nearly constant molecular density in assembled monolayers in our control experiments, which ensures that the differences in charge transport behavior are primarily governed by the changes in energetics of the charge transport channels rather than the differences in peptide packing density. We performed X-ray photoelectron spectroscopy (XPS) to estimate molecular packing density in assembled peptides.^49^Figure 2c–f show results from XPS characterization of PHH SAMs with and without heme, and XPS results for other peptide sequences are shown in Supporting Information, Figures S13–S16. These results show that elemental composition is consistent with peptide sequence designs, and in cases of heme incorporation, Fe is detected in the SAMs. Interestingly, our XPS analysis revealed two sulfur signals—one consistent with sulfur covalently bound to gold and the other corresponding to disulfide bonds.^50^ These results suggest that a small fraction of peptides may not be covalently linked to the gold surface, despite the formation of a densely packed monolayer consisting of peptides covalently linked to the gold surface.

We next determined a relative comparison of molecular packing density across different samples by determining areas under the curves from XPS spectra, which avoids complications from determining absolute molecular densities due to X-ray attenuation and chemical complexity in adsorbed molecular layers.^51^ Each peptide chain contains only 1 S atom per molecule (in the cysteine residue). Therefore, we calculated the area under the Au 4f and S 2p XPS curves and used the ratio of S: Au as a characteristic quantity of peptide molecules on gold surfaces. Here, the S: Au ratio was 0.006 for PHH SAMs without heme in peptide-containing solutions and 0.005 for PHH SAMs incubated with heme in solution during monolayer preparation. These results suggest that the molecular packing density remains relatively constant in the presence or absence of heme. Therefore, we expect that differences in electron transport between heme-free and heme-containing peptide monolayers should arise due to heme doping rather than differences in peptide packing density.

### 2.3 Peptide Conductivity Is Significantly Enhanced upon Heme Loading

**Figure 3:**
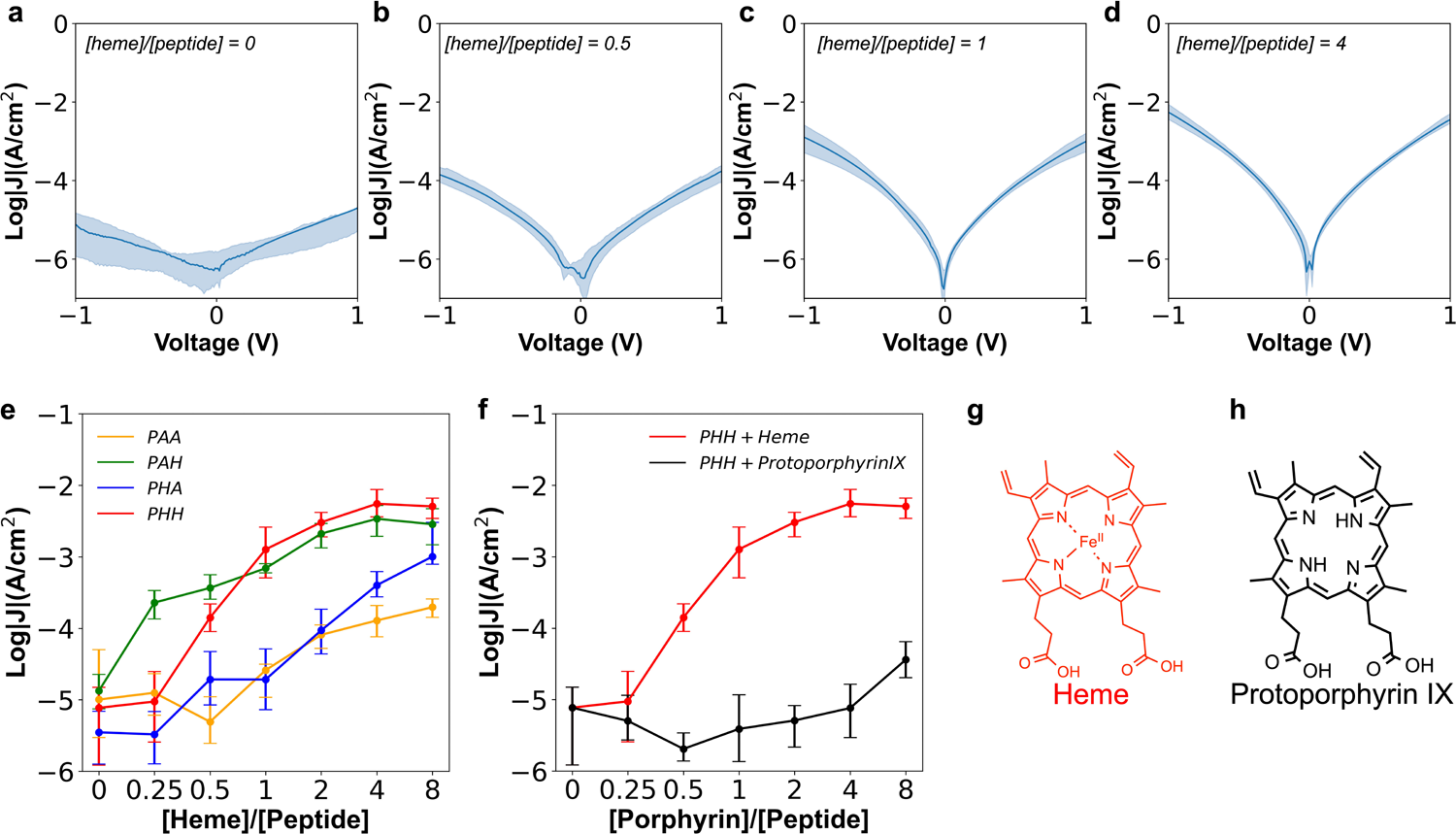
Electronic characterization of peptide monolayers prepared using different heme-to-peptide ratios in solution. (a–d) Current density–voltage response curves for PHH monolayers for different heme to peptide ratios. The solid line is the average current–voltage response over >70 individual scans from multiple distinct contact areas across the peptide SAM, and the shaded area denotes the 25–75 percentile range of the data. (e) Absolute current density of peptide monolayers at −1 V applied bias as a function of the heme to peptide ratio during SAM preparation. (f) Absolute current density of PHH monolayers with heme or protoporphyrin as a function of heme to peptide ratio during SAM preparation in solution. (g) Chemical structure of heme. (h) Chemical structure of protoporphyrin IX.

We next characterized the electronic properties of peptide SAMs using a liquid metal soft contact technique known as EGaIn.^35^ In this method, conical tips consisting of liquid metal (eutectic gallium indium, EGaIn) serve as the top contact and a template-stripped gold substrate (Au^TS^) serves as the bottom contact.^35^ Current density–voltage (*I*–*V*) curves are determined by sweeping the applied bias from 0 V to +1 V, then to −1 V, and finally back to 0 V, while measuring current from >70 scans at more than 7 locations across SAMs. Current density (units of A/cm^2^) is determined by directly measuring the area of the liquid metal contact using a camera during the measurements. To understand how the current density changes with heme loading, we systematically prepared peptide SAMs at different heme-to-peptide ratios in solution, as shown in Figure 3 and Supporting Information Section 7.4.

In the absence of heme, the *I*–*V* curves for pristine PHH monolayers show relatively low absolute current density (Figure 3a). This observation is consistent with prior work on pure peptide monolayers with ≈3 nm thickness,^52^ which likely occurs because the heme-free peptides lack electronically active cofactors that could enhance electronic charge transport. As the heme concentration is increased during SAM formation, the current density begins to rise (Figure 3b–d). A significant enhancement in current density is observed as the heme concentration is increased above a heme-to-peptide ratio of 0.5. Interestingly, the current density at −1 V increases by >3 orders of magnitude at a heme-to-peptide ratio of 8 compared to peptide SAMs without heme.

Beyond a heme-to-peptide ratio of 8, measurements are limited by the solubility of heme in aqueous solution. Given the consistent thickness and packing density of SAMs, our results suggest that the significant increase in current density arises due to the incorporation of heme into peptide monolayers. We note that for PHH monolayers, the significant enhancement in current density occurs above a heme-to-peptide ratio of 0.5 during SAM preparation in solution and saturates around a heme-to-peptide ratio of 4, whereas the UV–vis titration suggests the saturation binding ratio in free solution is around 0.9 (Supporting Information, Figure S5d). These results suggest that a higher concentration of heme is required to drive the saturation of heme binding on peptide monolayers on gold surfaces compared to peptides in free solution. This observation can be rationalized by considering cooperative effects originating in densely packed monolayers such as peptide-heme interactions, peptide–peptide interactions, crowding effects, and site-blocking effects.^53−55^

We next investigated the charge transport mechanisms in peptide monolayers. In electronic junctions with nanometer-scale gap dimensions, electron transport generally occurs through two distinct mechanisms: tunneling or hopping. Tunneling is a quantum mechanical phenomenon that involves electrons passing through an energy barrier without occupying intermediate energy levels.^56^ In contrast, hopping relies on electrons sequentially transiting between localized molecular sites and is driven by thermal activation.^57^ Temperature-dependent measurements are commonly used to gain insights into the underlying charge transport mechanisms in mesoscale systems.^58^ However, conducting such experiments generally requires additional fabrication relying on cooling chambers and microfluidic devices.^59,60^ Instead, we adopted an alternative approach by analyzing experimental data using analytical expressions for current–voltage (*I*–*V*) relations from the tunneling and hopping models (Supporting Information Section 7.5). In hopping-dominated transport, the current–voltage response arises from charge transfer rates *R* governed by Marcus theory, which account for the reorganization energy describing changes to the system and surrounding environment to facilitate electron hopping.^61^ The hopping current is given by
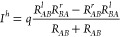
1where *I*^*h*^ is the hopping current, *R*_*AB*_^*l*^ is the rate at the left electrode at which electrons transfer from the electrode to the molecule, *R*_*BA*_^*r*^ is the rate at the right electrode at which electrons transfer from the molecule to the electrode, and *q* is the elementary charge. In tunneling-dominated transport, the current–voltage response is described by Landauer formula, which accounts for the transmission probability governed by molecule-electrode coupling γ, energy level differences Δ*ε*, and state densities.^62^ The tunneling current is given by
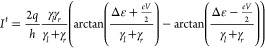
2where *I*^*t*^ is the tunneling current, *h* is Planck’s constant, γ_*l*_ is the molecule-electrode coupling for the left electrode, γ_*r*_ is the molecule-electrode coupling for the right electrode, and *eV* is the applied voltage multiplied by the elementary charge. Prior work reported that the experimentally measured current in systems governed by hopping transport spans a much broader range of current values compared to tunneling-dominated transport.^63^ Motivated by these findings, we analyzed experimental data using single-level models for tunneling and hopping transport as reported in prior literature (Supporting Information Section 7.5) using eqs 1 and 2 to model hopping and tunneling-dominated transport, respectively.^63^ These functional forms determine the shape of *I*–*V* curves and were used to fit our experimental *I*–*V* data from EGaIn measurements. Our results show that the experimentally determined *I*–*V* curves for heme-bound peptides are in good agreement with hopping model, whereas the tunneling model fails to qualitatively capture the observed behavior for these materials. Conversely, the experimental *I*–*V* curves for peptide monolayers in the absence of heme show good agreement with the tunneling model (Supporting Information, Figure S18). These results suggest that the addition of heme introduces hopping sites for electron transport, enabling dramatic increases in current density.

To understand the role of peptide sequence on the electronic properties of SAM junctions, we performed *I*–*V* measurements using the EGaIn technique for several different peptides: PAA, PAH, PHA, and PHH (Figure 3e and Supporting Information, Figure S17). Results from these experiments are summarized by plotting current density for each sequence at −1 V as a function of heme-to-peptide ratio (Figure 3e). Our results show that the current density at −1 V increases for all peptide SAMs as the heme concentration is increased, albeit to different extents depending on peptide sequence. Current density also increases upon increasing heme concentration for peptide sequence PAA, which lacks histidine residues for specific heme coordination. Although the increase in current density for PAA SAMs at high heme-to-peptide ratios is smaller compared to sequences containing histidine, these results suggest some amount of nonspecific binding of heme in peptide monolayers, which likely arises due to hydrophobic interactions.^10^ Interestingly, only a single amino acid substitution of histidine for alanine in the peptide sequence PAA (compared to PAH) resulted in a significant enhancement in the current density at moderate heme loading. Although all peptide sequences showed enhanced current densities upon increasing heme-to-peptide ratios, the increase in current differed substantially despite minor changes in amino acid sequences. Notably, peptide sequences PAH and PHH exhibited more substantial current density increases at lower heme-to-peptide ratios before plateauing, whereas PHA and PAA generally showed more gradual increases at moderate concentration ratios. At intermediate heme-to-peptide ratios (e.g., [heme]:[peptide] ≈ 1–2), current density varied by 2 orders of magnitude for different peptide sequences (e.g., PHH or PAH versus PHA or PAA), which highlights the strong dependence of electronic properties on peptide sequence.

We further aimed to understand the role of the metal ion in heme cofactors on the electronic properties of peptide SAMs. A series of EGaIn experiments was performed using protoporphyrin IX (Figure 3h), a molecule structurally similar to heme (Figure 3g) but lacking the central iron atom required for coordination with histidine (Figure 3f). Peptide SAMs with the PHH sequence containing protoporphyrin IX exhibited no significant increase in current density at −1 V at low to moderate porphyrin-to-peptide ratios, compared to the same sequence with similar heme-to-peptide ratios. These findings highlight that specific coordination with the central iron atom is essential for achieving large enhancements in current within peptide monolayers. We also considered the role of amino acid sequence and the approach for heme incorporation during SAM formation.^64^ For the results presented in this work, peptide SAMs were first prepared on gold surfaces, followed by addition of heme to the aqueous solution containing the SAM. This approach was necessary to minimize nonspecific adsorption of heme on bare gold surfaces in the absence of peptide SAMs. Our results suggest that the efficiency of heme binding in peptide SAMs is influenced by the amino acid sequence, particularly the position of the coordinating histidine residue required for binding to heme. In densely packed monolayers with the N-terminal cysteine anchored to gold, sequences PHH and PAH feature a solvent-exposed histidine, readily accessible for coordination. In contrast, the histidine in sequence PHA is buried deeper within the monolayer, potentially hindering heme penetration and interaction with the histidine. This explains our experimental observation that PHA behaves similarly to PAA at low heme-to-peptide ratios. Additionally, PHH does not exhibit a significantly higher current density than PAH, despite containing two histidine residues and a theoretically greater binding capacity for heme. To further explore the heme-binding properties of these peptide SAMs, we employed molecular modeling and simulation.

### 2.4 Molecular Modeling and Simulations

**Figure 4:**
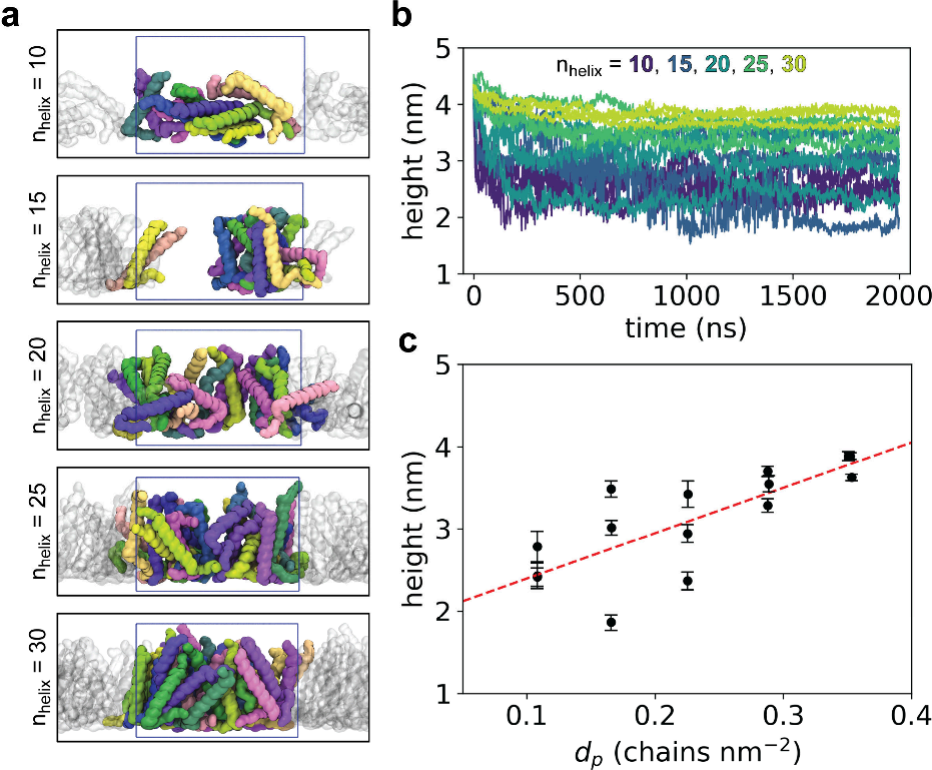
MD simulations of peptide SAMs reveal an interdependence between monolayer height and packing density. (a) Side-view snapshots of assembled peptide monolayers after 2 μs restrained MD. One replica of each *n*_*helix*_ condition is shown. The periodic unit cell is depicted as a blue box. Each peptide chain is shown as a brightly colored surface and its periodic images are shown as gray transparent surfaces. (b) Height versus time for each simulation replica of each *n*_*helix*_ condition. Inset text shows color legend. (c) Weighted least-squares regression of height (*h*) versus packing density (*d*_*p*_). Scatter points and error bars indicate mean ± st.dev of height over the last 500 ns of each simulation replica. Red dashed line indicates WLS regression line. Inset red text indicates WLS parameter values ± parameter variance.

We began by using molecular dynamics (MD) simulations to understand the role of molecular packing density on monolayer thickness. Peptide monolayers were modeled across a wide range of lateral packing densities using a two-step approach. In the first step, initial monolayer structures were generated using Packmol^65^ rigid-body packing with rationally guided spatial packing constraints (Supporting Information, Figures S19–S22). These molecular assemblies were modeled at various packing densities consisting of 10, 15, 20, 25, or 30 peptide chains within a 10 nm × 10 nm area (Figure 4a). In all cases, three independent replicas of each packing condition were simulated. In the second step, MD simulations were used to study the structure and dynamics of these molecular assemblies. Two additional restraining potentials were applied during the simulations to implicitly represent interactions between the gold substrate and peptides, including a harmonic potential to anchor the peptide’s N-terminal thiol sulfur to the implicit substrate and an excluded volume potential to prevent peptide diffusion into the surface of the implicit substrate (Supporting Information, Figure S23). After 2 μs of equilibration with restraints, monolayer thickness and packing density are calculated and plotted as a function of time (Figure 4b, Supporting Information, Figures S24–S28). The average thickness (or height) of each monolayer in the terminal 500 ns of simulation is then determined and plotted as a function of packing density (Figure 4c). Weighted least-squares regression was performed using the mean and variance of height versus mean packing density over the final 500 ns of each simulation. Our results show that monolayer height strongly depends on packing density, such that denser packing gives rise to thicker monolayers. Interestingly, smaller packing densities give rise to a broader height distribution, as demonstrated by the convergence between multiple independent simulation replicas at higher packing densities. Using the weighted least-squares regression line, the structural properties of PHH monolayers are determined to have a height *h* = 3.03 nm and packing density *d*_*p*_ = 0.215 chains nm^–2^.

A series of MD simulations was then performed to gain additional insight into the heme penetration and diffusion process into peptide monolayers at atomic-scale resolution. First, a 30-helix monolayer of PHH peptides was prepared following the procedure described above. Several point mutations were then incorporated on the histidine residues of the assembled PHH monolayer to create PAH, PHA, and PAA monolayers with identical initial assembled structures.^66^ A solution of 30 heme molecules was then placed above each of these four monolayer systems (PHH, PAH, PHA, and PAA), and the resulting systems were subjected to MD simulation to allow diffusion and infiltration of heme into peptide monolayers. Three independent replicas were prepared for each peptide sequence with randomized heme placement. A series of restraints was applied to protein and heme during the heme diffusion process, and sulfur-restraining and excluded volume potentials were similarly maintained during heme diffusion. A substrate excluded volume potential was also applied to heme molecules to prevent diffusion through the lower periodic boundary. An additional excluded volume potential acting on heme was also placed near the top of the simulation box to prevent diffusion of heme through the upper periodic boundary, ensuring that heme only penetrates the monolayer from the upper solution phase. Finally, a Morse potential was added between every iron atom of heme and every *ε*-nitrogen atom of histidine to allow for reversible coordination bond formation between heme and histidine.^67^ Current strategies for parametrizing heme-histidine coordination in the CHARMM36m force field only allow for harmonic bonds between the iron atom of heme and the ε-nitrogen atom of histidine. To allow for reversible ligation of heme by histidine, we introduce the Morse potential, which locally acts as a harmonic bond at small distances and dissipates at larger interatomic distances. A schematic illustration of the spatial restraints utilized during heme diffusion simulations is shown in Supporting Information, Figure S29.

During the initial round of simulations, heme molecules were observed to aggregate in the solution directly above the monolayer before interacting with and penetrating the peptide monolayer, which occurred due to the hydrophobic nature of heme molecules. To prevent heme aggregation, we implement an antiaggregation potential by modifying the energy associated with heme–heme nonbonded Lennard-Jones interactions using NBFix parameters, following a prior example in the literature for preventing aggregation of hydrophobic membrane permeants.^68^ Here, the value of ϵ_*ij*_ is set to zero for all intermolecular interactions between π-conjugated heavy atoms of heme, eliminating heme’s tendency to aggregate without compromising interactions with protein or solvent. A series of control simulations of heme solutions was performed with and without NBFix parameters, showing that NBFix significantly attenuates heme aggregation (Supporting Information, Figure S30).

**Figure 5:**
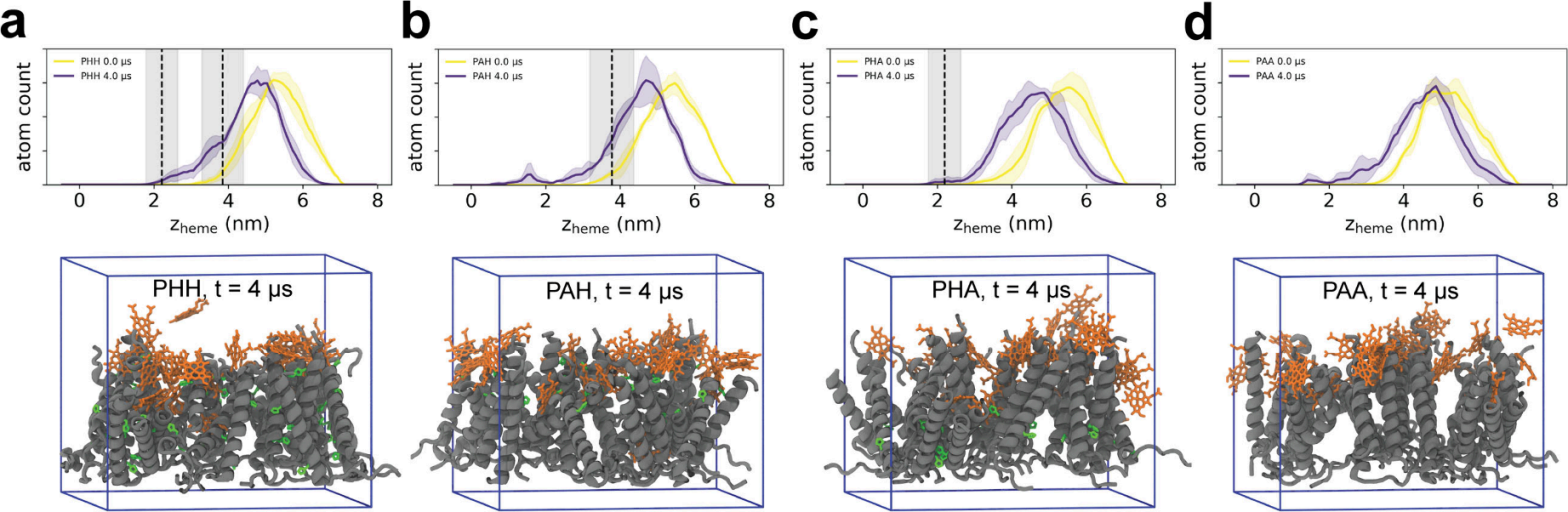
MD simulation results for heme permeation into (a) PHH, (b) PAH, (c) PHA, and (d) PAA peptide monolayers. Top row shows replica-averaged distributions ± 1 SD (standard deviation) for heme heavy-atom *z*-positions as a function of time. Average *z*-position ± 1 SD for histidine shown as vertical black dashed lines and shaded gray areas. Bottom row shows snapshots of heme penetration into peptide monolayers after 4 μs simulation with heme molecules shown in orange, peptide molecules in gray, and histidine side chains in green.

Using this approach, three independent replicas of each peptide monolayer sequence were simulated under heme diffusion conditions for 4 μs each. The resulting heme permeation profiles are shown in Figure 5 and Supporting Information, Figure S31. Our results show that heme slowly infiltrates peptide monolayers as a function of time and forms coordination bonds with histidine residues. In PHH and PAH monolayers, shallow histidine residues (H29) located near the solution phase are the first to form coordination bonds with heme, occurring within the first hundred nanoseconds of simulation, and these bonds persist and proliferate throughout the 4 μs of simulation. In contrast, deeply buried histidine residues at position 15 (sequences PHH, PHA) seldom form coordination bonds with heme molecules, reflecting the sluggish dynamics controlling heme penetration into peptide monolayers within these simulation time scales (Supporting Information, Figure S32). Notably, histidine coordination events occur reversibly, indicating that heme molecules are mobile such that permeation into the monolayer occurs within MD simulation time scales.

Heme permeation profiles (Figure 5a–d; Supporting Information, Figure S31) highlight the influence of peptide sequence on heme transport into monolayers. Regardless of peptide sequence, several heme molecules tend to reside around *z* = 4 nm, which is slightly above the peptide monolayer. A few heme molecules are observed to deeply penetrate into the peptide monolayer (Supporting Information, Figure S34), but the entire process of heme infiltration occurs slowly, generally beyond the time scales captured by our MD simulations. Nonspecific contacts between peptides and heme rapidly increase and plateau over the course of simulation, with similar nonspecific contacts observed across all four sequences (Supporting Information, Figure S33).

Atomic-scale resolution MD simulations demonstrate that heme permeation into peptide membranes is an inherently stochastic process driven by competing networks of interactions. Performing multiple replicas of each simulation aids in accounting for the stochastic nature of these processes and quantifying the uncertainty associated with the heme transport. However, due to the demanding nature of atomic-scale resolution MD simulations, only 4 μs of simulation could be performed per peptide sequence and per simulation replica in this work. Further modeling studies could take advantage of coarse-grained protein force fields to accelerate sampling of underlying molecular processes. However, the difference between experimental and computational time scales should be noted. In our experiments, heme diffusion into peptide membranes is allowed to proceed for 24 h, significantly longer (by a factor of ∼ 10^11^) than the 4 μs simulations reported here. Though the apparent difference in time scales can be attenuated by strategies such as modified nonbonded potentials via NBFix to eliminate heme aggregation, MD simulation of such slow diffusive processes remains a challenging task in the field. Therefore, alternative methods could be useful in interrogating the heme permeation process, particularly by using enhanced sampling methods,^69,70^ to recover free energy landscapes of heme insertion into the self-assembled monolayer. Such methods could provide new and additional insight into the molecular mechanisms governing slow dynamic phenomena and for characterizing the self-assembly behavior of bioelectronic materials.

## 3 Conclusions

This study demonstrates the potential of sequence-defined, heme-binding peptides to enhance electron transport in thin films. By integrating heme units with custom-designed peptides, peptide SAMs were developed with significantly enhanced electron transport properties. Our results reveal a >1000-fold increase in current density across the peptide SAM junctions upon heme loading, as measured using liquid metal alloy soft contacts, without altering the film thickness. This substantial enhancement in conductivity underscores the importance of metal-binding cofactors, such as heme, in facilitating electron transport within biomolecular materials. In addition, the ability to modulate electron transport through variations in amino acid composition and sequence highlights the versatility and tunability of peptide-based materials.

This work offers a prototype for developing new bioelectronic materials by providing new routes to design and engineer electronically conductive peptides. Our work shows that specific amino acid composition, sequences, and heme loading levels allow for control over the electronic conductivity of peptide-based biomaterials. These materials could be used in cell-on-chip systems and tissue engineering applications (e.g., neural cell-electrode interfaces), where a biocompatible and conductive interfacial layer is required for efficient signal transmission. The amino acid sequences reported in this work were originally inspired by cytochrome *bc*_1_, which further highlights the role of sequence design. In future work, a broad library of sequences can be explored to further expand the functionality and versatility of these materials. Moving forward, understanding the underlying sequence-structure–function relations governing these materials will facilitate the development of new molecular design principles and enable development of practical peptide-based bioelectronic materials.

## Supporting Information Available

More information about experimental details, materials, characterization, methods and analysis (PDF)
