# Supplementary material for: Amino Acid Sequence Controls Enhanced Electron Transport in Heme-Binding Peptide Monolayers
Source: ACS Cent Sci. 2025 Apr 2;11(4):612–21. doi: 10.1021/acscentsci.4c01849 (PMC12022913; doi:10.1021/acscentsci.4c01849)
Supplement: Supplementary file 1 — oc4c01849_si_001.pdf [file oc4c01849_si_001.pdf]

Supplementary Information

Amino Acid Sequence Controls Enhanced Electron Transport in Heme-Binding Peptide Monolayers

Hao Yang[1,2†], Xiaolin Liu,[1,3†], Moeen Meigooni[1,4†], Li Zhang[5,6], Jitong Ren[1,7], Qian Chen[1,2,3,7,8], Mark Losego[5,6], Emad Tajkhorshid[1,3,4,9*], Jeffrey S. Moore[1,2,3*], Charles M. Schroeder[1,2,3,4,7*]

[1]Beckman Institute for Advanced Science and Technology, University of Illinois at Urbana-Champaign, Urbana, IL, 61801, USA

[2]Department of Materials Science and Engineering, University of Illinois at Urbana-Champaign, IL, 61801, USA

[3]Department of Chemistry, University of Illinois at Urbana-Champaign, Urbana, IL, 61801, USA

[4]Center for Biophysics and Quantitative Biology, University of Illinois Urbana-Champaign, Urbana, IL 61801, USA

[5]School of Materials Science and Engineering, Georgia Institute of Technology, Atlanta, GA 30332, USA

[6]Renewable Bioproducts Institute, Georgia Institute of Technology, Atlanta, GA, 30332, USA

[7]Department of Chemical and Biomolecular Engineering, University of Illinois Urbana-Champaign, Urbana, IL, 61801 USA

[8]Chan Zuckerberg Biohub Chicago, Chicago, IL 60642, USA

[9]Department of Biochemistry, University of Illinois at Urbana-Champaign, Urbana, IL 61801, USA

Table of Contents

1 Materials and Synthesis 3

2 Circular Dichroism (CD) Experiments 6

3 UV-vis Titration Experiments 7

4 Atomic Force Microscopy 10

5 ATR-FTIR 15

6 X-ray Photoelectron Spectroscopy 16

7 EGaIn Measurements 20

8 Molecular Dynamics Simulations 26

1 Materials and Synthesis

Reagents for peptide synthesis were purchased from Sigma-Aldrich. CEM was the vendor for all Fmoc-protected amino acids and Cl-MPA ProTide^{TM} (LL) resin. A Liberty Blue^{TM} system (CEM Corp.) with solid-phase Fmoc chemistry was used to synthesize all peptides. All syntheses were carried out at the 0.05 mmol scale in excess of a 0.2 M amino acid in dimethylformamide (DMF) solution and in the presence of 0.5 M *N*,*N*'-diisopropylcarbodiimide (in DMF) and 0.5 M ethyl (hydroxyimino)cyanoacetate (oxyma, in DMF). A 20% v/v of piperazine in DMF was used as the deprotection solution for the removal of Fmoc-protecting groups. Post-reaction, the resin was separated and washed with dichloromethane (DCM) followed by DMF. The peptides were cleaved from the resin using a cleavage cocktail (trifluoroacetic acid (TFA)/triisopropylsilane (TIS)/H_2O/3,6-dioxa-1,8-octanedithiol (DODT) at a ratio of 92.5/2.5/2.5/2.5 v/v/v/v) at room temperature over a period of 3 hours. After this duration, the solution was filtered and the filtrate was added to diethyl ether, resulting in the formation of a crude peptide precipitate. The crude peptides were purified via preparative Waters Binary preparative high-performance liquid chromatography (HPLC) using a XBridge Prep C18 5μm OBD column. The mobile phase consisted of a gradient over 60 minutes of 25% H_2O with 0.1% TFA and 75% acetonitrile with 0.1% TFA (0 min) until 45% H_2O with 0.1% TFA and 55% acetonitrile with 0.1% TFA (60 min). The pure fractions were reunited and lyophilized (Labconco). Analytical HPLC spectra were determined on a Shimadzu HPLC instrument consisting of a Shimadzu LC-20AD pump with DGU 20A degassing unit, a Shimadzu SIL-20AHT autosampler, a CBM 20A communication module, and a SPD-M20A PDA detector. HPLC experiments were performed in isocratic mode using a Shimadzu Nexcol C18 column 5 μm (50×3.0 mm) at 25°C with absorbance detection at 230 nm. MALDI-TOF mass spectrometry experiments were performed using a Bruker Daltonics UltrafleXtreme MALDI TOF/TOF.

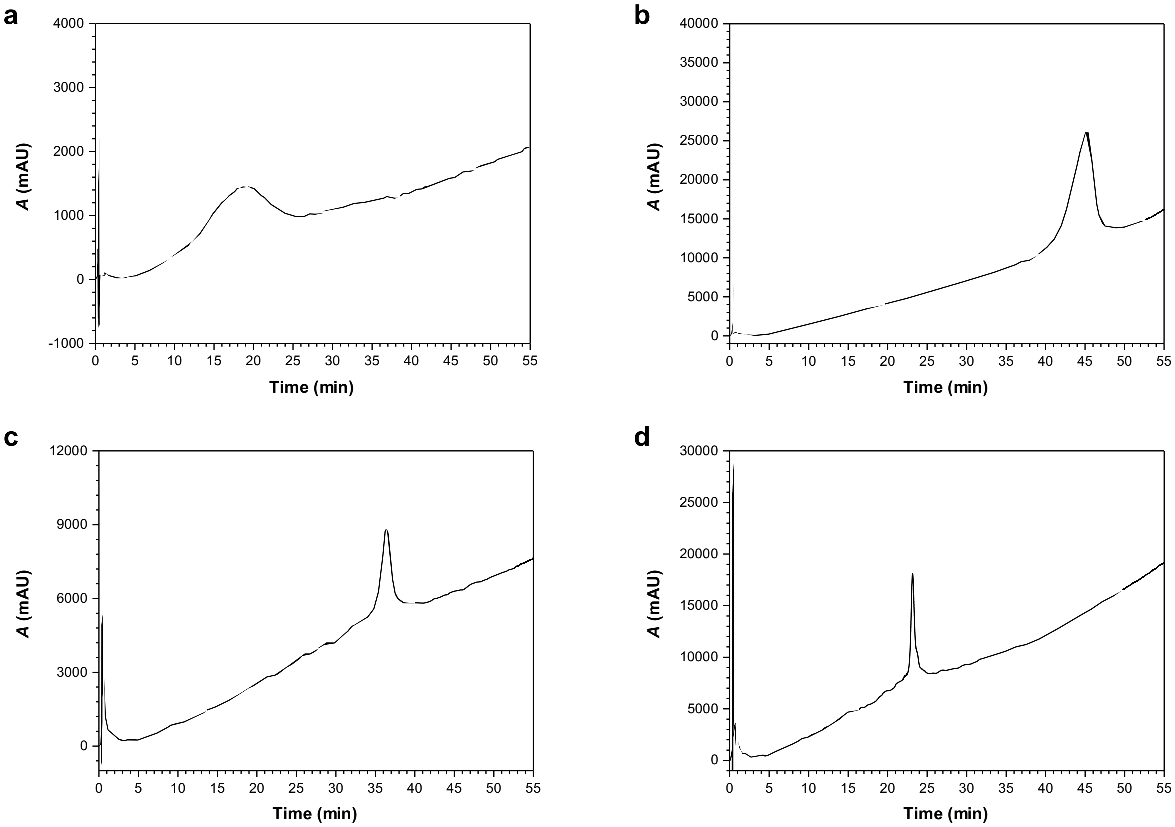

Figure S1. Analytical HPLC results for (a) PAA, (b), PAH, (c) PHA, and (d) PHH. HPLC experiments were performed using a C18 column with a gradient elution method, with the ratio of acetonitrile (containing 0.1% v/v TFA) to water (containing 0.1% v/v TFA) increasing from 25% to 45% over 55 minutes at a flow rate of 0.8 mL/min. The absorbance detector wavelength was 230 nm.

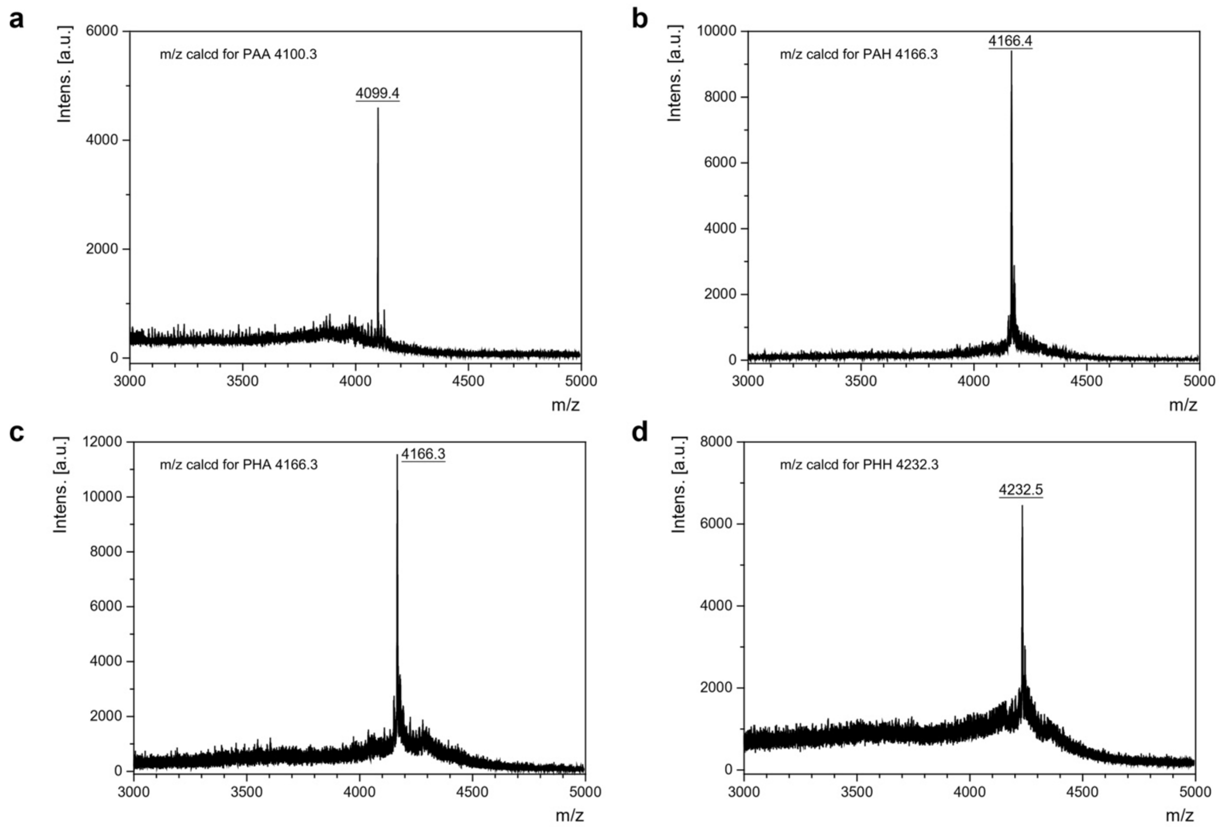

Figure S2. MALDI-TOF mass spectrometry characterization of peptide sequences (a) PAA, (b) PAH, (c) PHA, and (d) PHH.

2 Circular Dichroism (CD) Experiments

Circular dichroism (CD) measurements were performed to characterize peptide secondary structure using a Jasco J1500 spectropolarimeter. CD spectra were collected from 190 nm to 270 at 0.2 nm intervals, a rate of 50 nm/min, a response time of 2 s, and a bandwidth of 1 nm. 2 mm path length quartz cuvettes were used for solution samples. To inhibit disulfide bond formation, 10 equivalents of a reducing agent tris(2-carboxyethyl)phosphine (TCEP) were added to the peptide solutions.

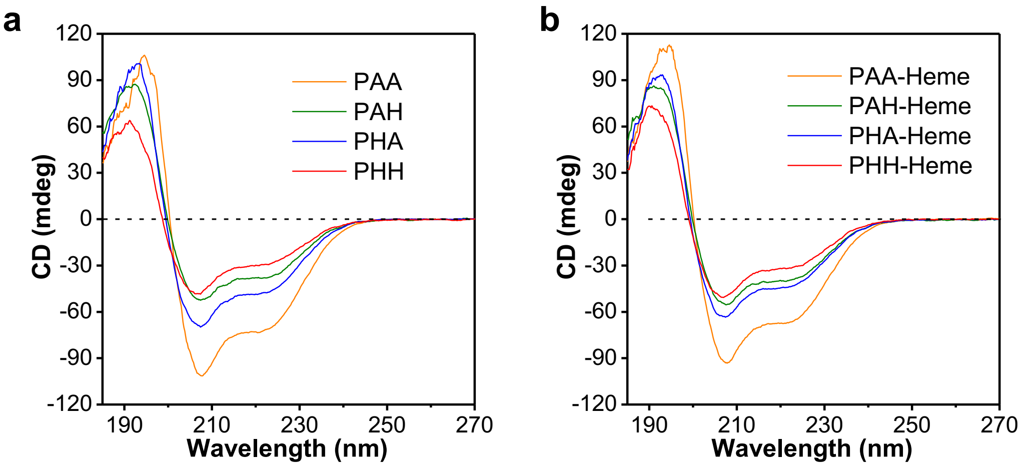

Figure S3: Circular dichroism (CD) spectra of the peptide sequences PAA, PAH, PHA, and PHH in the presence and absence of heme. The peptide concentration was 0.1 mg/mL, with molar ratios of heme to peptide as follows: [Heme]:[PAA] = 1:1, [Heme]:[PAH] = 1:1, [Heme]:[PHA] = 1:1, and [Heme]:[PHH] = 2:1.

Table S1. Deconvolution analysis of the peptide sequences PAA, PAH, PHA, and PHH in the presence and absence of heme using DichroWeb software.

	Helix 1	Helix 2	Strand 1	Strand 2	Turns	Unordered
PAA	0.861	0.139	0	0	0	0
PAA-Heme	0.766	0.234	0	0	0	0
PAH	0.829	0.171	0	0	0	0
PAH-Heme	0.802	0.198	0	0	0	0
PHA	0.796	0.204	0	0	0	0
PHA-Heme	0.781	0.219	0	0	0	0
PHH	0.739	0.261	0	0	0	0
PHH-Heme	0.777	0.223	0	0	0	0

The deconvolution results indicate that the CD signals are predominantly attributed to alpha-helical structures, with no evidence of a random coil contribution.

3 UV-vis Titration Experiments

For the UV-vis titration experiments, a 200 μM peptide stock solution was prepared in phosphate-buffered saline (1X, pH 7.4) (PBS), and a 2 mM stock hemin solution was prepared in dimethyl sulfoxide (DMSO). Titrations were performed by incremental addition of the hemin stock solution to the peptide solutions, while adjusting the volume of PBS to maintain a constant total volume and ensure a consistent peptide concentration. The peptide concentration was maintained at 20 μM throughout the experiments. In addition, 10 equivalents of TCEP were included to prevent disulfide bond formation. Absorption measurements were obtained on an Agilent Cary 60 UV-Vis spectrophotometer, using a standard 1 cm path length quartz cuvette.

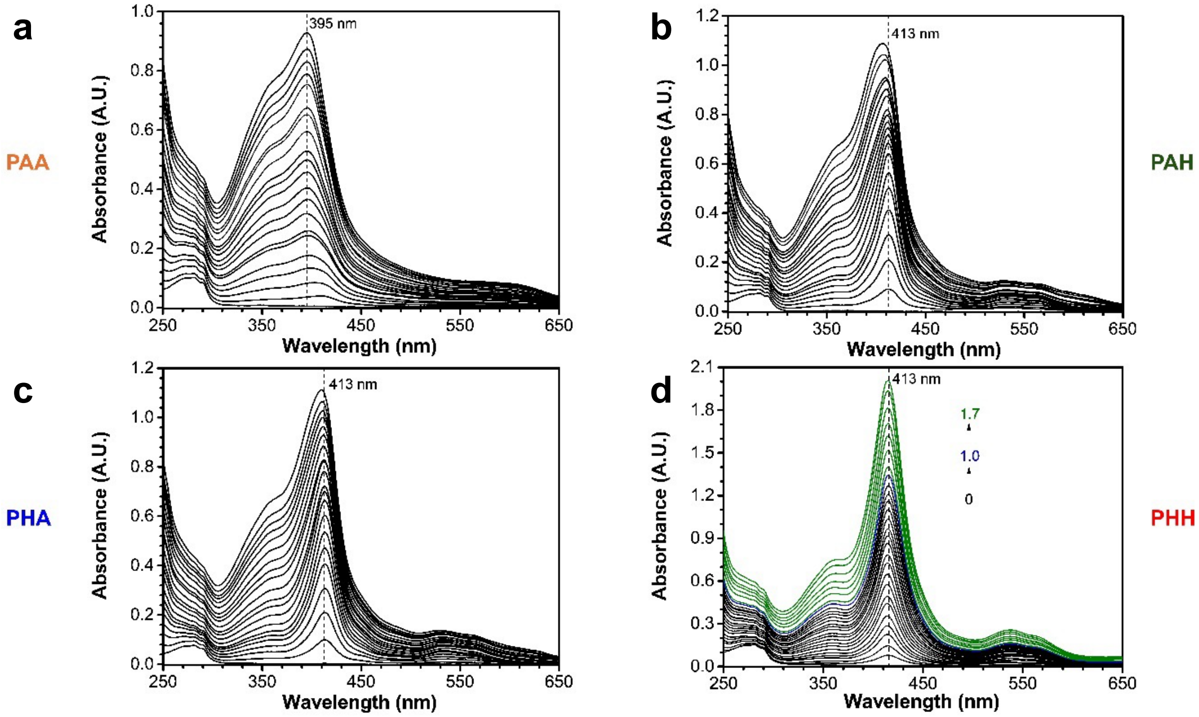

Figure S4: Absorption spectra obtained for peptide sequences PAA, PAH, PHA, and PHH upon incremental addition of hemin stock solution. (a–c) UV-vis spectra of heme titrations into a 20 μM solution of (a) PAA, (b) PAH, and (c) PHA determined in a 1 cm path length quartz cuvette, upon addition of 0, 0.05, 0.1, 0.15, 0.2, 0.25, 0.3, 0.35, 0.4, 0.45, 0.5, 0.55, 0.6, 0.65, 0.7, 0.75, 0.8, 0.85, 0.9, 0.95, and 1 equiv of heme per peptide sequence. (d) UV-vis spectra of heme titrations into a 20 μM solution of PHH determined in a 1 cm path length quartz cuvette, upon addition of 0, 0.05, 0.1, 0.15, 0.2, 0.25, 0.3, 0.35, 0.4, 0.45, 0.5, 0.55, 0.6, 0.65, 0.7, 0.75, 0.8, 0.85, 0.9, 0.95, 1, 1.1, 1.2, 1.3, 1.4, 1.5, 1.6, and 1.7 equiv of heme per peptide sequence.

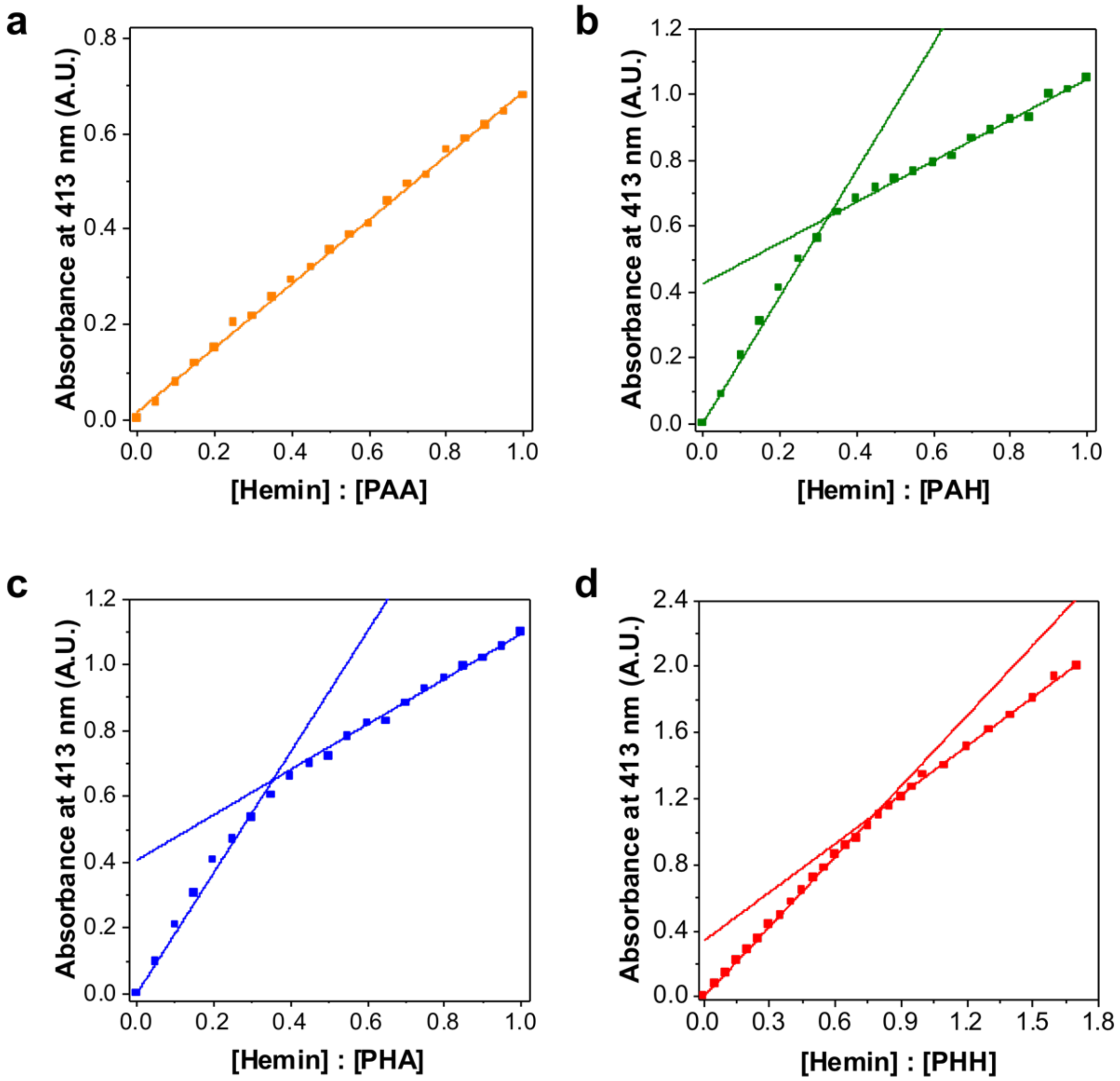

Figure S5: Peak absorbance values at 413 nm plotted as a function of the [hemin]:[peptide] ratio for (a) PAA, (b) PAH, (c) PHA, and (d) PHH. Solid lines show a linear regression fit to the experimental data. The [hemin]:[peptide] ratio at which the slope changes in each data set corresponds to the number of specifically bound hemins that each peptide sequence can bind before saturation.

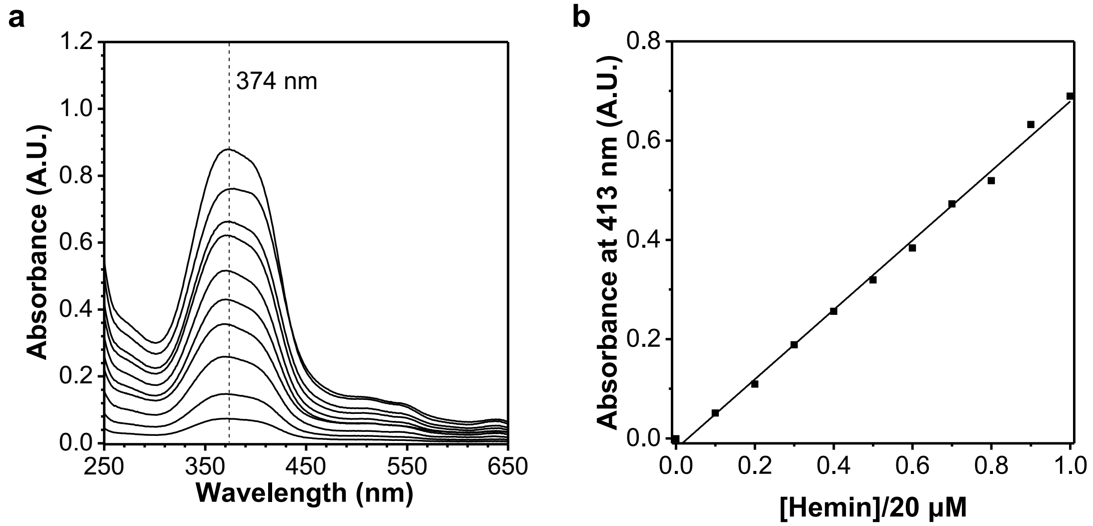

Figure S6. UV-vis absorption spectra obtained during hemin titration. (a) Spectra recorded in a 1 cm path length quartz cuvette with the sequential addition of hemin in 2 μM increments to a 20 μM solution. (b) Peak absorbance values at 413 nm plotted as a function of the hemin concentration. Solid lines show a linear regression fit to the experimental data.

4 Atomic Force Microscopy

Atomic force microscopy (AFM) was used to characterize monolayer morphology as well as monolayer thickness. The surface topography was measured in tapping mode using an Asylum Research MFP-3D AFM and Tap300Al-G AFM probes. A central area of 1 μm^2 was scratched using the AFM tip to determine monolayer thickness. Prior to the AFM tip 'scratching' procedure, force calibration was performed for the AFM probes. We first performed a control experiment by applying 200 nN of force using the AFM tip, which showed no significant damage to the Au surface (**Figure S7**). Subsequently, peptide monolayers were scratched under contact mode by applying a constant force of 100 nN. To visualize the step change in profile height after removal of adsorbed peptide from the central area, section profiles were constructed by drawing a horizontal band spanning across both the scratched and unscratched regions. For numerical thickness determination, topography height histograms were determined utilizing data from topography pixels. These data were analyzed by applying two Gaussian fits to this height histogram, allowing determination of the separation distance between the peak positions from the fitted Gaussian curves.

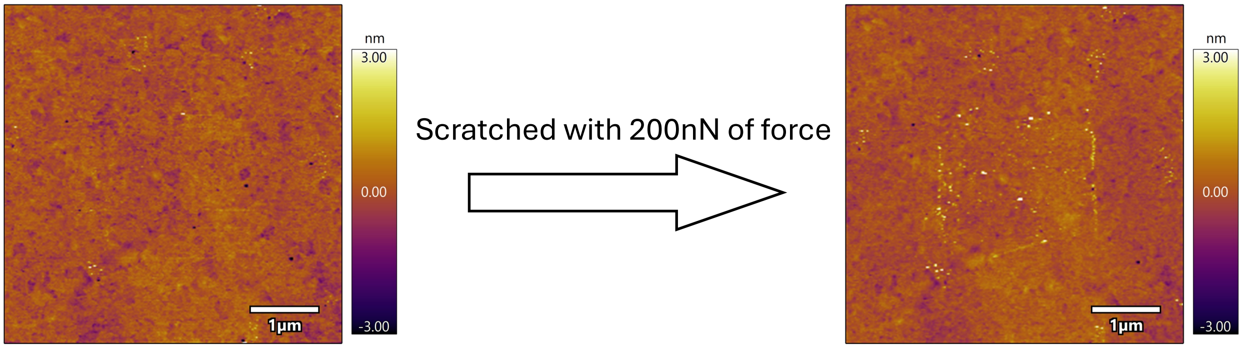

Figure S7. Surface topography of bare Au^{TS} before and after scratching with an AFM tip at 200 nN of applied force.

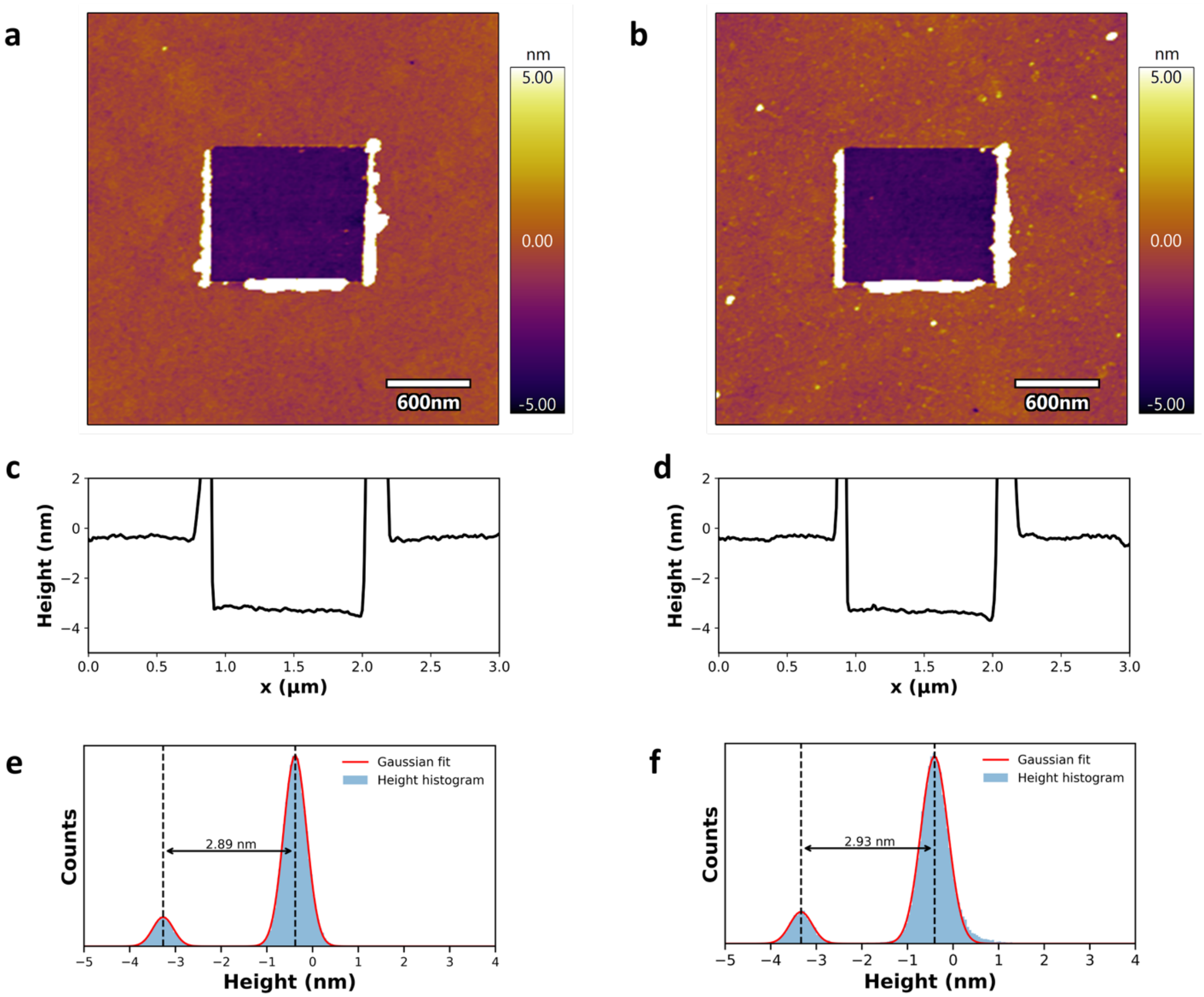

Figure S8. AFM topography of peptide monolayers for (a) PAA peptides in the absence of heme, (b) PAA peptides in the presence of heme. Section profile of AFM topography for (c) PAA peptides in the absence of heme and (d) PAA peptides in the presence of heme. Height histograms of AFM topography for (e) PAA peptides in the absence of heme and (d) PAA peptides in the presence of heme.

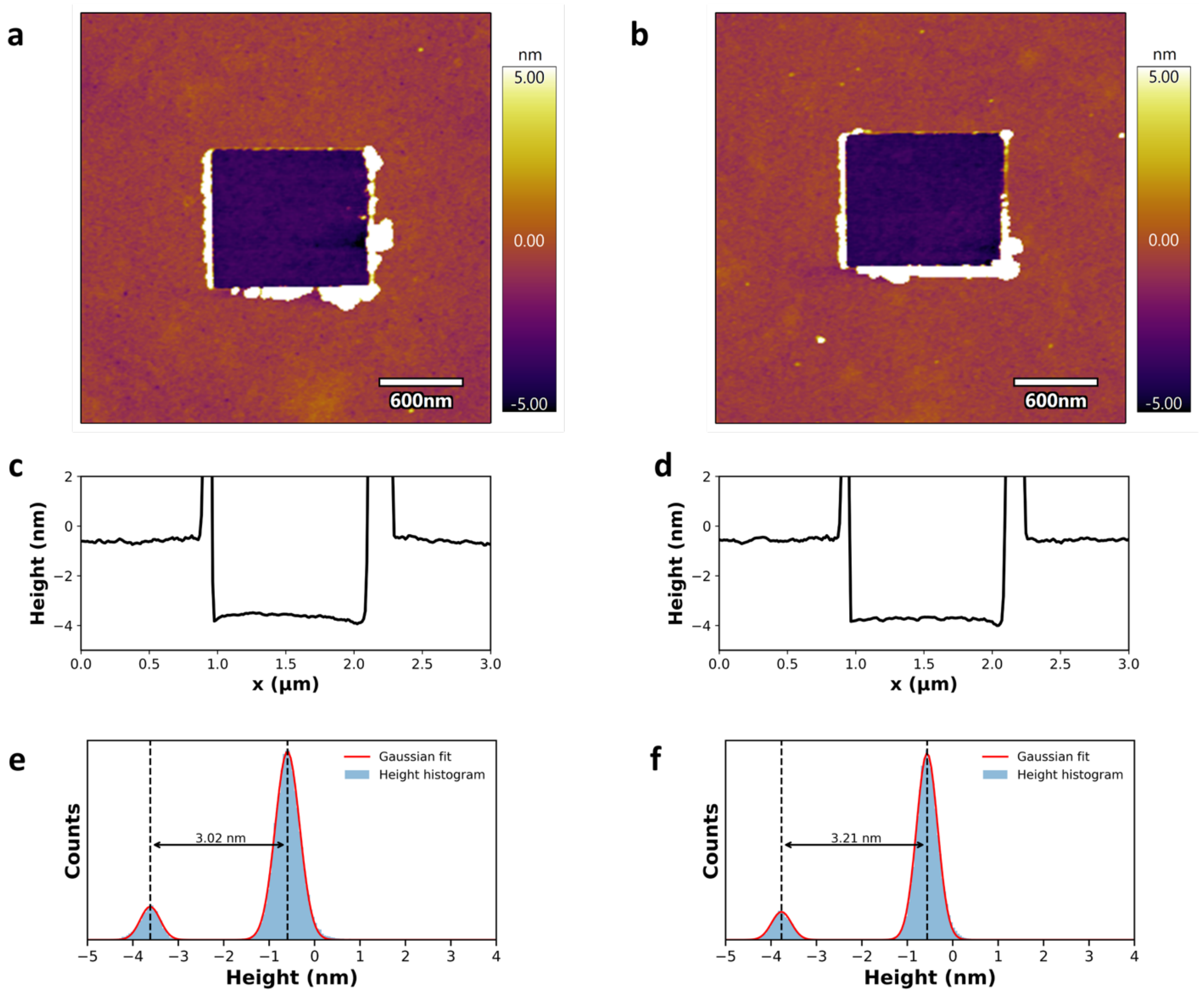

Figure S9. AFM topography of peptide monolayers for (a) PHA peptides in the absence of heme and (b) PHA peptides in the presence of heme. Section profile of AFM topography for (c) PHA peptides in the absence of heme and (d) PHA peptides in the presence of heme. Height histograms of AFM topography for (e) PHA peptides in the absence of heme and (d) PHA peptides in the presence of heme.

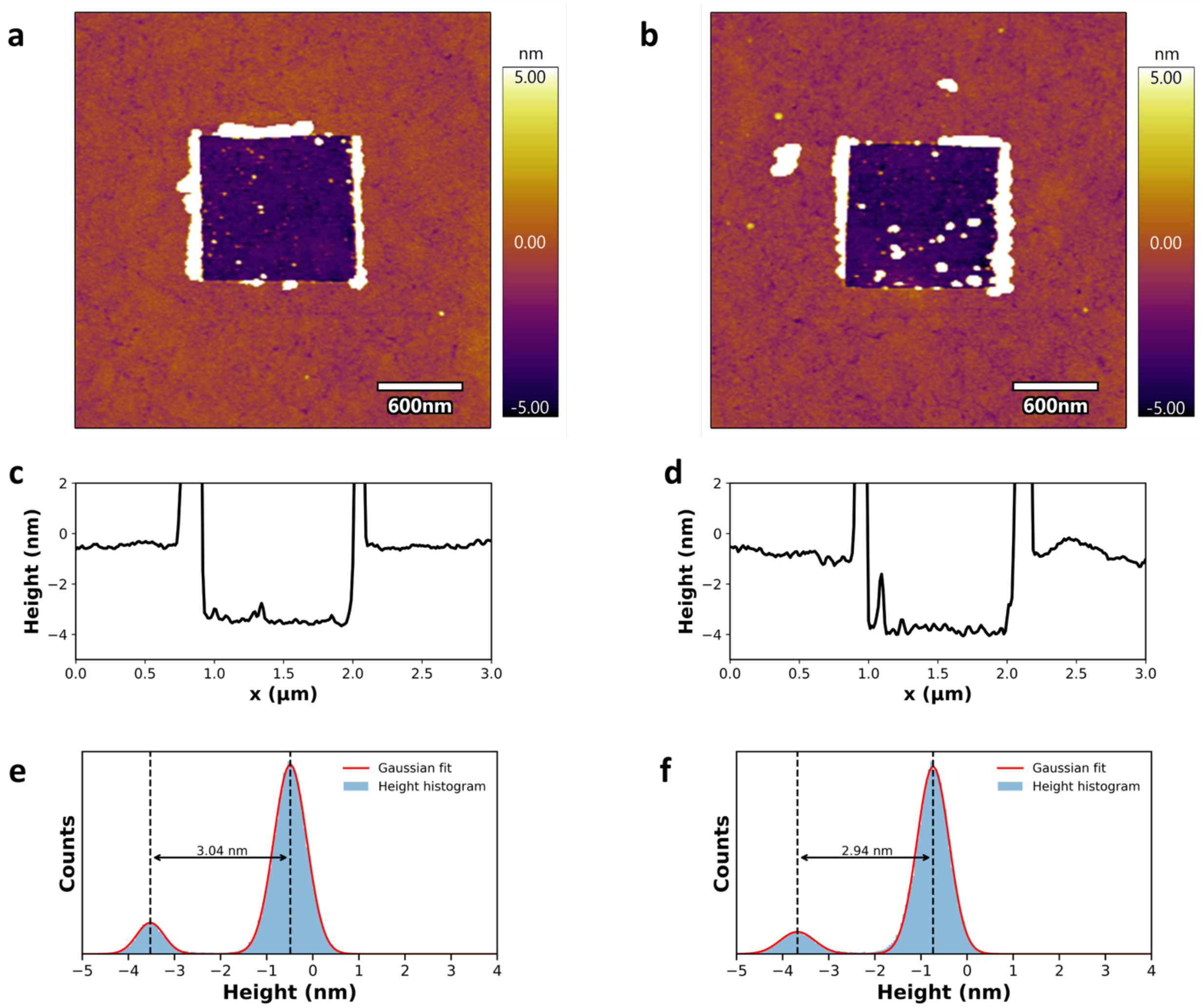

Figure S10. AFM topography of peptide monolayers for (a) PAH peptides in the absence of heme, (b) PAH peptides in the presence of heme. Section profile of AFM topography for (c) PAH peptides in the absence of heme and (d) PAH peptides in the presence of heme. Height histograms of AFM topography for (e) PAH peptides in the absence of heme and (d) PAH peptides in the presence of heme.

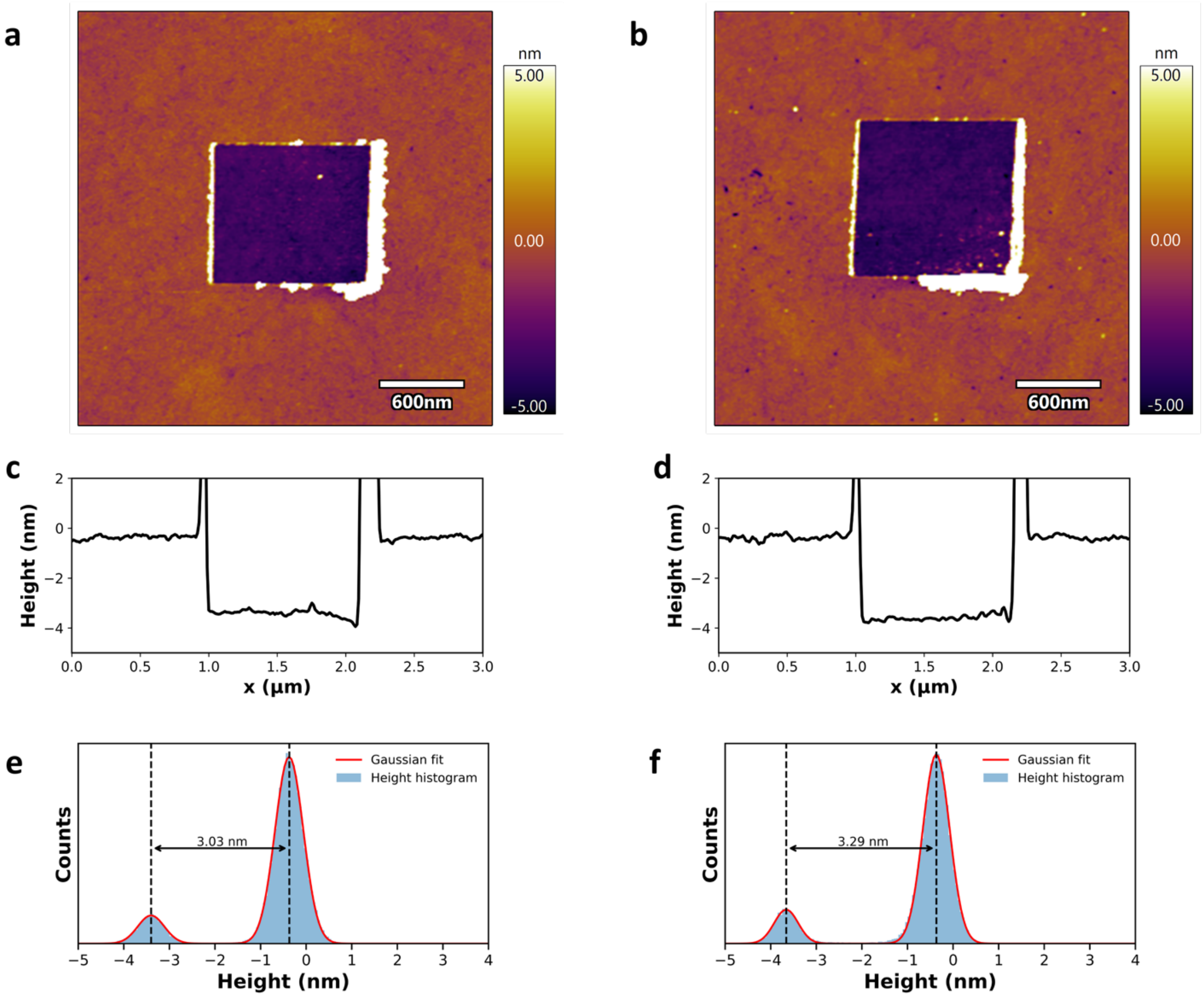

Figure S11. AFM topography of peptide monolayers for (a) PHH peptides in the absence of heme, (b) PHH peptides in the presence of heme. Section profile of AFM topography for (c) PHH peptides in the absence of heme and (d) PHH peptides in the presence of heme. Height histograms of AFM topography for (e) PHH peptides in the absence of heme and (d) PHH peptides in the presence of heme.

5 ATR-FTIR characterization

Attenuated total reflectance-Fourier transform infrared (ATR-FTIR) spectroscopy was performed using a Thermo Nicolet iS50 FTIR to evaluate the secondary structure of peptides on a gold surface. For the dried peptide measurement, a peptide solution (PAH) was first deposited onto the ATR crystal, and the solvent was allowed to fully evaporate. ATR-FTIR analysis was then performed on the solid peptide on the surface. For measurements involving gold substrates (bare Au and peptide self-assembled monolayers (SAMs) on gold surfaces), the substrate was pressed directly onto the ATR crystal for data collection, allowing for identification of amide bond peaks.

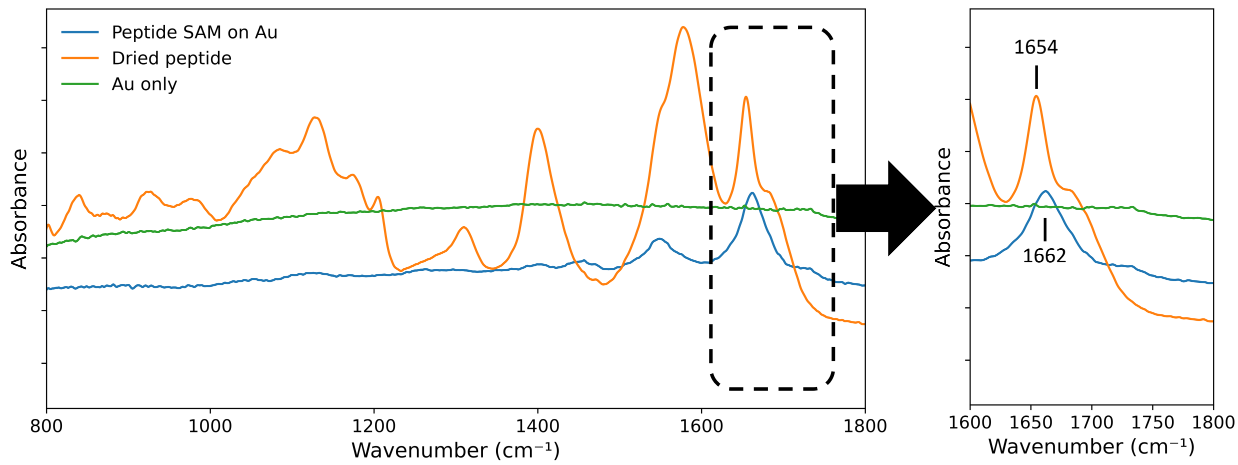

Figure S12. ATR-FTIR spectra of peptide monolayers (PAH) on gold surfaces and dried peptide samples (PAH), highlighting the Amide I peak region. The inset provides a zoomed-in view of the Amide I peak.

6 X-ray Photoelectron Spectroscopy

XPS was performed using a Thermo Scientific K-α system with a monochromatic Al K-α X-ray source (1487 eV) with a 60° incident angle and 90° emission collection angle. High resolution scans were collected at a 0.1 eV step size. The Au $4f_{7/2}$ peak (84.0 eV) was used as the charge reference for analysis. Measurements were made within 1 week of material synthesis to minimize and prevent oxidation of the peptide chains. Data were analyzed using a Shirley background for all spectra with a Lorentzian/Gaussian mix of 30%. Several results were interpreted from S spectra, so S doublets were fit such that FWHM of both $2p_{1/2}$ and $2p_{3/2}$ peaks were equivalent, peak areas were $2p_{3/2}$:$2p_{1/2}$ = 2:1, and the difference in binding energy was 1.2 +/- 0.1 eV. Au doublets were fit such that FWHM of both peaks were equivalent, peak areas were $4f_{7/2}$:$4f_{5/2}$ = 4/3 +/- 5% and the difference in binding energy was 3.7 +/- 0.5 eV. Although fitted Fe peaks are shown, low Fe concentration in the samples prevents specific binding energies from being determined. Instead, Fe spectra had their background subtracted and peaks fitted only to show the presence of Fe and approximate binding energies (+/- 1 eV)

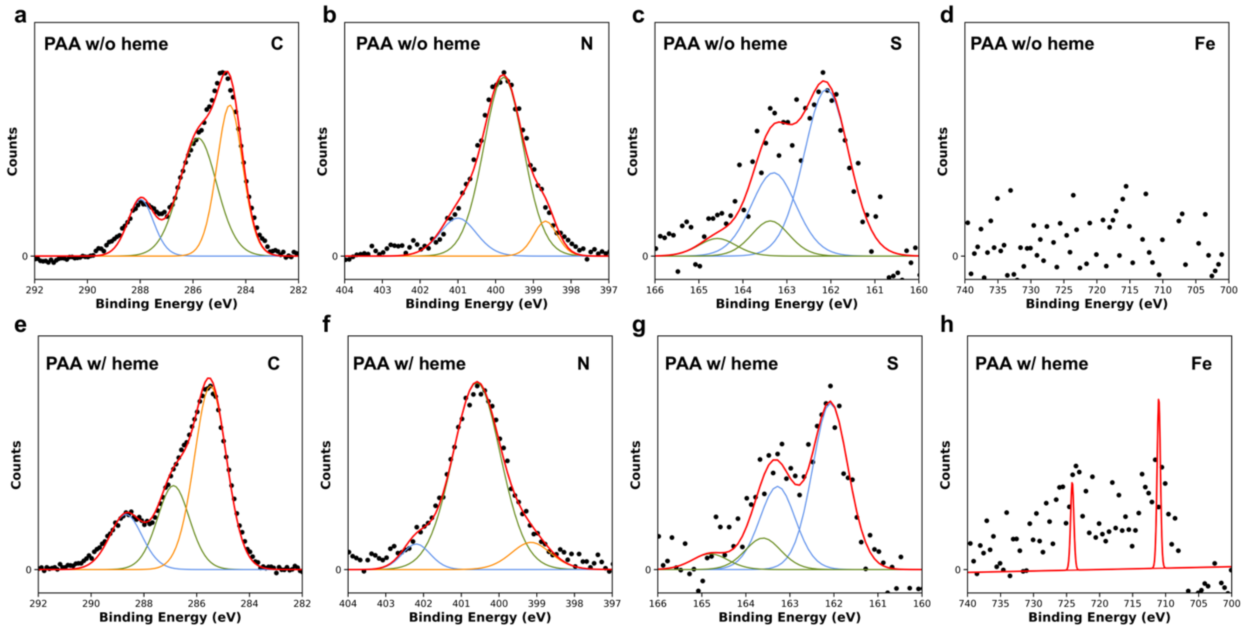

Figure S13. High-resolution XPS spectra for (a-d) PAA monolayers without heme and (e-h) with heme during SAM preparation. Elemental spectra are shown for (a,e) C 1s, (b,f) N 1s, (c,g) S 2p, and (d,h) Fe 2p with raw data points (black points), appropriate deconvolutions (yellow, green and blue), and overall fit (red).

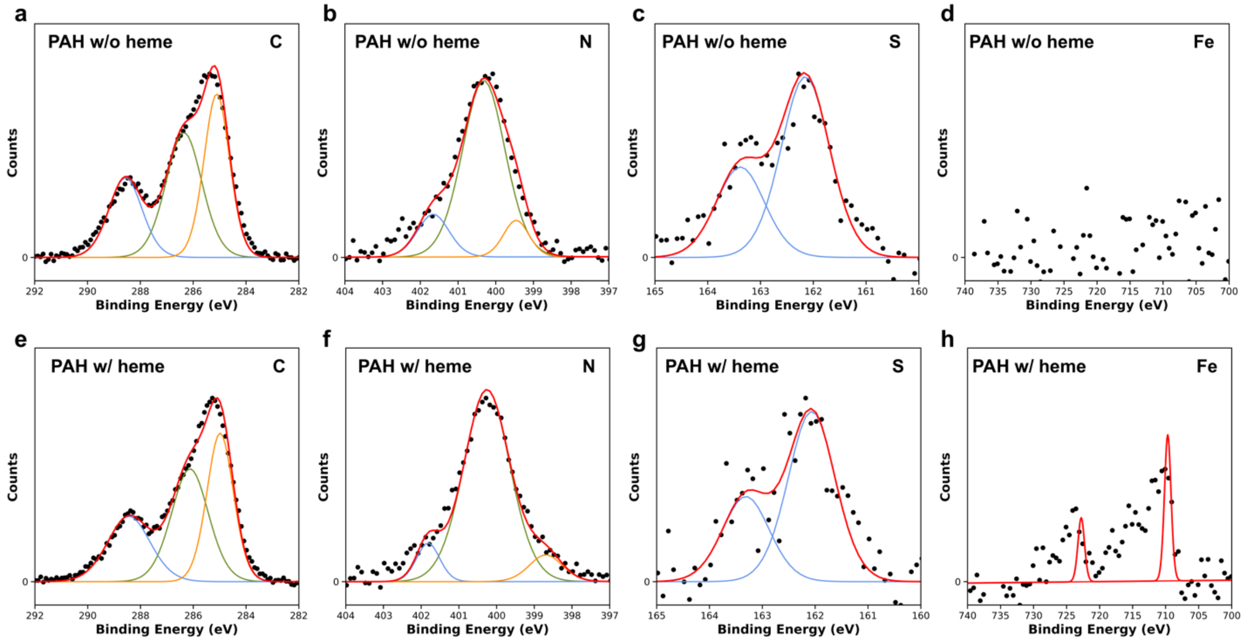

Figure S14. High-resolution XPS spectra for (a-d) PAH monolayers without heme and (e-h) with heme during SAM preparation. Elemental spectra are shown for (a,e) C 1s, (b,f) N 1s, (c,g) S 2p, and (d,h) Fe 2p with raw data points (black points), appropriate deconvolutions (yellow, green and blue), and overall fit (red).

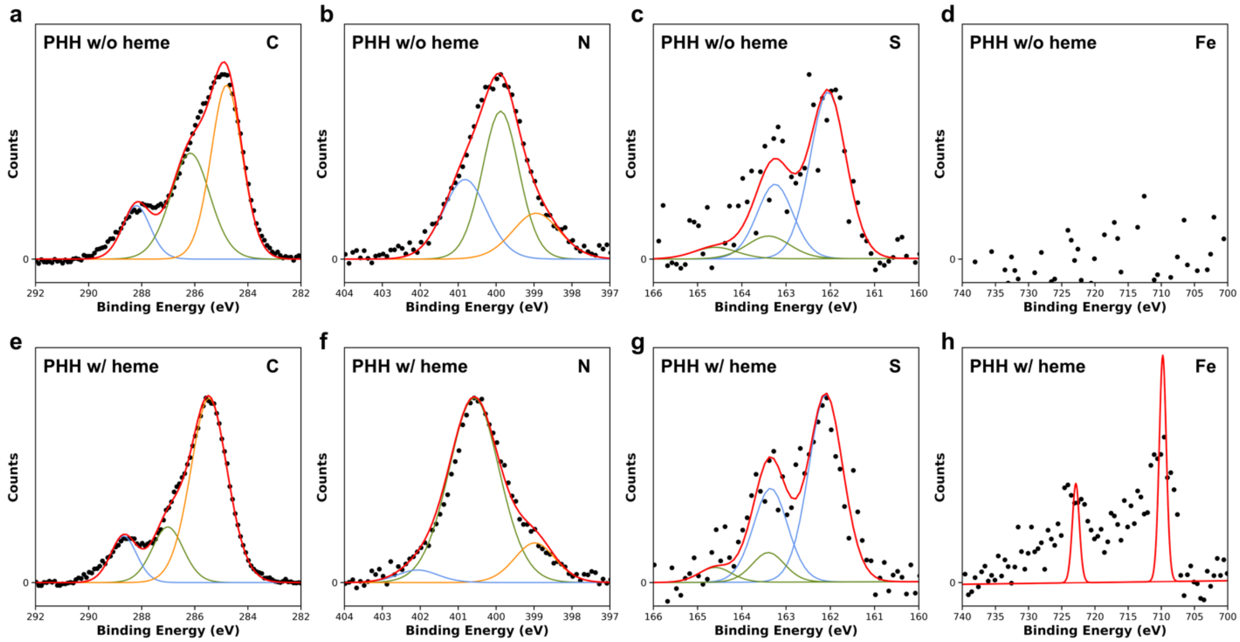

Figure S15. High-resolution XPS spectra for (a-d) PHH monolayers without heme and (e-h) with heme during SAM preparation. Elemental spectra are shown for (a,e) C 1s, (b,f) N 1s, (c,g) S 2p, and (d,h) Fe 2p with raw data points (black points), appropriate deconvolutions (yellow, green and blue), and overall fit (red).

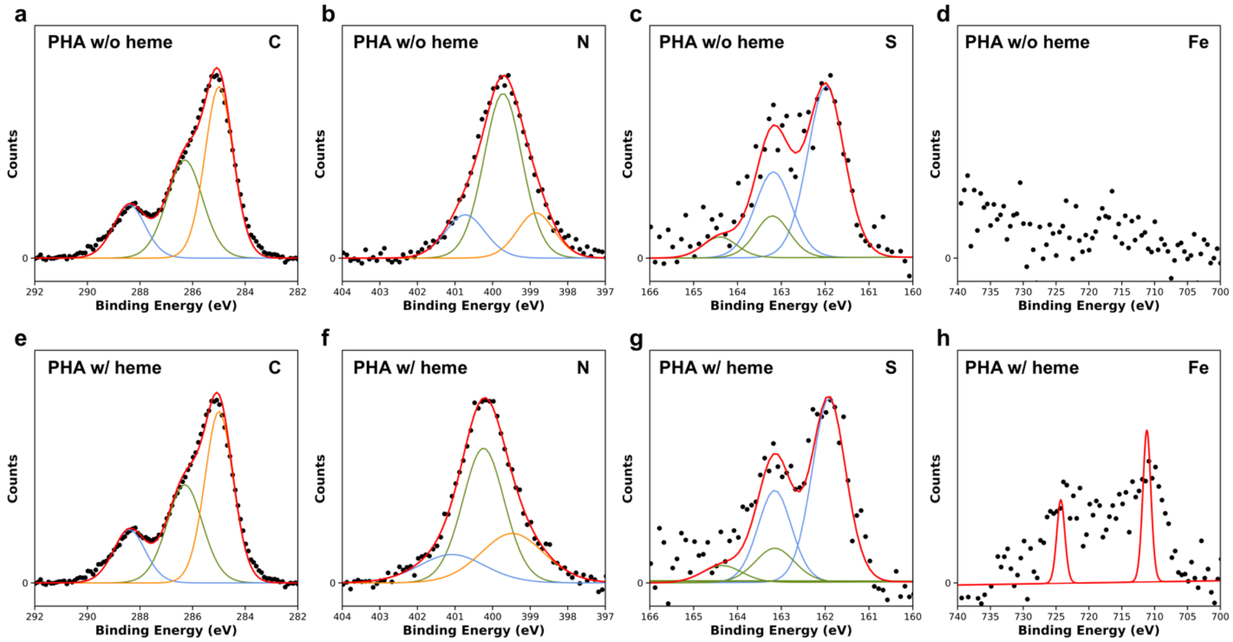

Figure S16. High-resolution XPS spectra for (a-d) PHA monolayers without heme and (e-h) with heme during SAM preparation. Elemental spectra are shown for (a,e) C 1s, (b,f) N 1s, (c,g) S 2p, and (d,h) Fe 2p with raw data points (black points), appropriate deconvolutions (yellow, green and blue), and overall fit (red).

7 EGaIn Measurements

7.1 Template striped gold surface

We use template striped gold surfaces (Au^{TS}) to prepare peptide SAMs owing to flatness of these surfaces (RMS roughness = 0.382 nm for a 5μm scan). For the fabrication of Au^{TS}, we start with clean silicon wafer. First, a standard silicon wafer cleaning process is performed, which involves sequential sonication in isopropyl alcohol, followed by acetone, and then again in isopropyl alcohol, with each step lasting 15 minutes. Next, a 200 nm layer of gold is deposited on the Si surface at a deposition rate of 0.5 Å/s using an E-beam evaporator. After the deposition, a droplet of Norland 61 optical adhesive is applied onto the gold surface, and a cleaned glass strip is placed on top of the adhesive. After the adhesive is cured, the gold layer is securely bonded to the glass surface. Prior to SAM growth, the glass strip is separated from the silicon wafer. Due to the poor adhesion between gold and silicon, the gold surface in contact with the silicon wafer is exposed. This surface, templated by the silicon wafer, is nearly atomically flat, providing an ideal substrate for SAM growth.

7.2 Monolayer preparation

We adopted a self-assembled monolayer (SAM) approach for monolayer preparation. Here, peptide samples are first dissolved in PBS solution at a concentration of 0.1 mg/mL. To ensure that the thiol group from the cysteine residue is reduced to allow for S-gold interaction, a reducing agent (0.5 mM TCEP) is added to solution to prevent disulfide bond formation. The Au^{TS} substrate was then placed in the peptide solution for SAM growth at a constant temperature of 4ºC.

For heme incorporation, an aliquot of a 5% stock solution of heme in DMSO is added directly into the peptide solution (for a target heme concentration), one hour after introducing the Au^{TS} substrate. This step allows for the formation of a peptide SAMs, followed by the incorporation of heme into peptide SAMs on the gold surface. Our results show that if hemin is added simultaneously with the peptide (or prior to addition of peptide), no peptide adsorption on gold was observed, as determined by AFM. This occurs because hemin has a strong affinity for gold and rapidly occupies adsorption sites, thereby preventing peptide binding to the surface. We therefore first allow the peptide to form a SAM on the gold surface before introducing hemin. The peptide binding process is relatively fast, and we allow a one-hour incubation period to ensure peptide binding on the electrode surface. The subsequent 24-hour period is primarily to allow hemin to diffuse into the peptide SAM to facilitate binding. Extending this incubation period

beyond 24 hours was found to be ineffective in additional changes or enhancements in conductivity. After 24 hours, the gold surface was removed from the peptide solution, rinsed with water, and dried using a gentle flow of nitrogen.

7.3 EGaIn//SAM/Au^{TS} Measurement

Electrical measurements were performed using a two-electrode configuration. The Au^{TS} served as the bottom electrode, while conical tips of eutectic gallium indium (EGaIn) alloy liquid metal were used as the top contact. The measurements were conducted in a nitrogen glovebox with an oxygen level controlled around 4% and relative humidity below 10%. The entire setup was constructed on a floated vibrationally isolated optical table to minimize mechanical instabilities. Liquid metal manipulation and contact establishment were executed using a Zaber motorized translation stage, which offers a resolution down to 0.03 µm/s.

For each SAM sample, at least seven different areas on the films were randomly chosen and sampled. At each spot, 10 potential sweeps were performed, following a sequence from 0 V to 1 V, then -1 V, and back to 0 V, with a step of 0.01 V and a scan rate of 0.005 s per step. The current-voltage relationship was measured using a Keithley 1602B source measuring unit. To determine the current density, the contact area was estimated based on the contact diameter of the conical tip. The results were compiled with no data selection, except for the exclusion of instances of short-circuit data where the current level rapidly approached the measuring limit of the instrument.

7.4 Current-voltage curves

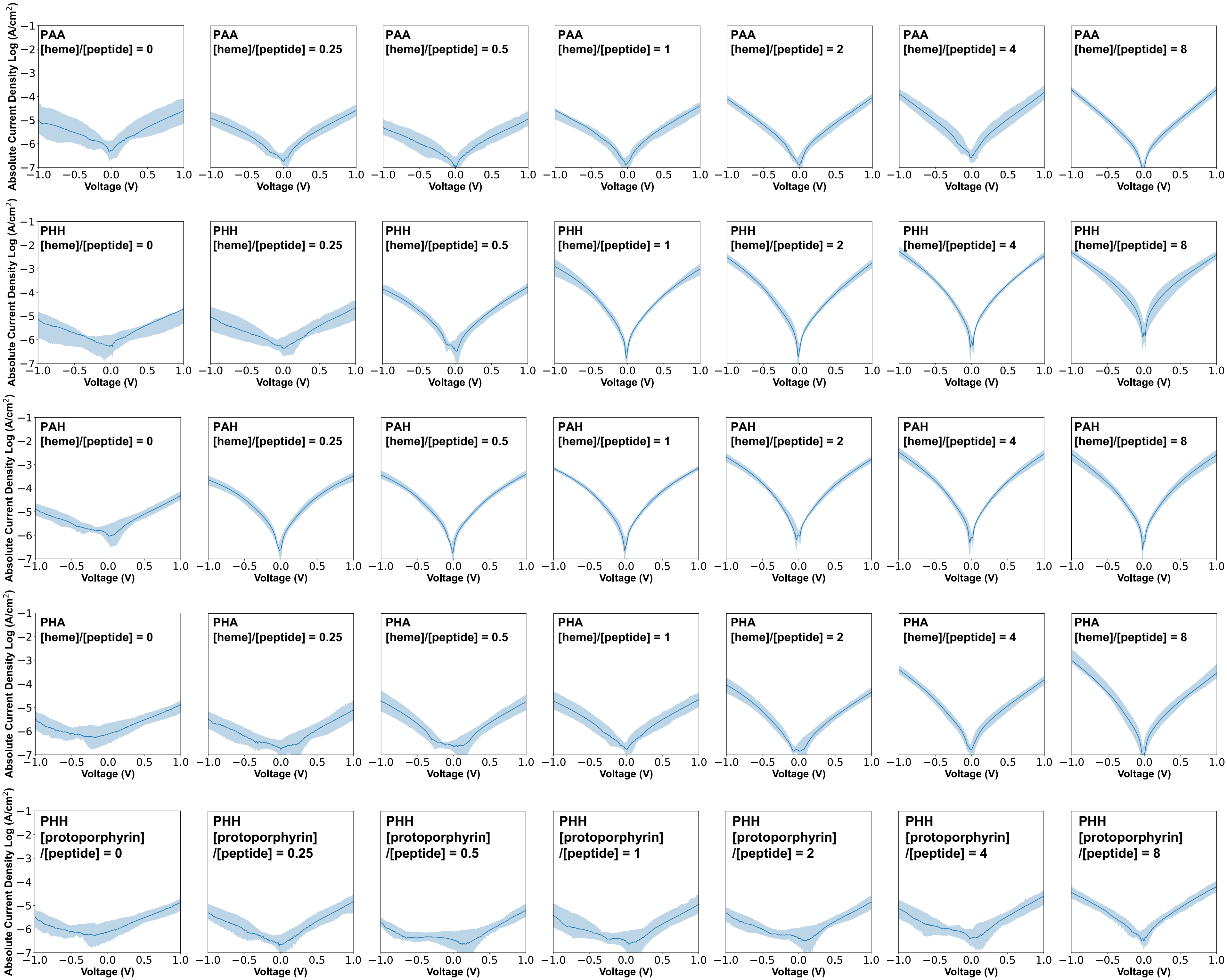

Figure S17. Current voltage curves for different peptide monolayers and SAM growth conditions. The [heme]/[peptide] or [protoporphyrin]/[peptide] ratio refers to the concentration ratio in solution during the self-assembled monolayer (SAM) growth process.

7.5 Current-voltage (I-V) relations and charge transport mechanisms

In electronic junctions with nanometer-scale dimensions, charge transport generally occurs by two mechanisms: tunneling or hopping. Tunneling is a quantum mechanical phenomenon that involves electrons passing through an energy barrier without occupying intermediate energy levels. In contrast, hopping relies on electrons sequentially transiting between localized molecular sites and is driven by thermal activation. The current-voltage (I-V) responses of tunneling and hopping-dominated transport exhibit distinct behavior due to their fundamental mechanistic differences. Here, we employed single-level models to quantitatively understand the I-V response for each transport mechanism.

Tunneling model

We used a simplified Simmons model for the current-voltage (I-V) response for the tunneling model. The Simmons model is a foundational approach to understand tunneling-dominated transport in molecular junctions, and it assumes a rectangular energy barrier and neglects the detailed chemical structure of the molecules. Within this framework, the tunneling current is described using the Landauer-Büttiker formula, which relates the current I to the transmission probability of electrons across the junction:[1]

$$I = \frac{2q}{h}\int [f_L(E) - f_R(E)]\,T(E)\,dE \tag{1}$$

where $f_L(E)$ and $f_R(E)$ are the Fermi distributions of electrons in left and right eletrodes, $T(E)$ is the transmission probability, which is given by:

$$T(E) = D_\varepsilon(E) 2\pi \frac{\gamma_L \gamma_R}{\gamma} \tag{2}$$

$$D_\varepsilon(E) = 2\pi \frac{\gamma/2\pi}{(E-\varepsilon)^2 + (\gamma/2)^2} \tag{3}$$

where $D_\varepsilon(E)$ is the broadened state density, ε is the state energy for the single level, $\gamma = \gamma_L + \gamma_R$ is the molecule-electrode coupling strength, and γ_L, γ_R are the coupling strengths of the molecule to the left and right electrodes, respectively. Using this framework, and assuming that the electron Fermi broadening of the electrodes is small and the Fermi distribution is approximated as a step function, the tunneling current I^t is given by:[2]

$$I^t = \frac{2q}{h}\frac{\gamma_L \gamma_R}{\gamma}\left(\arctan\left(\frac{\Delta\varepsilon + \frac{eV}{2}}{\gamma}\right) - \arctan\left(\frac{\Delta\varepsilon - \frac{eV}{2}}{\gamma}\right)\right) \tag{4}$$

where q is elementary charge, eV is elementary charge multiplied by the applied voltage, and h is Planck's constant. Equation 4 was used to plot and fit data to the tunneling model.

Hopping model

We constructed a single-level hopping model following a similar procedure as described in prior work.[2] In hopping-dominated transport, the current is determined by the charge transfer rate at the interace between molecules and electrodes following Marcus theory.[3]

$$R_{AB}^{K} = \int_{-\infty}^{+\infty} k_K(E) f_K(E) F(E-\varepsilon)\, dE \quad (5)$$

$$R_{BA}^{K} = \int_{-\infty}^{+\infty} k_K(E)[1-f_K(E)] F(\varepsilon - E)\, dE \quad (6)$$

where R_{AB}^{K} is the charge transfer rate, the superscript $K = L$ or R represents left or right electrode, subscripts AB and BA represent electron transfer from the electrode to the molecule or from the molecule to the electrode, respectively, k is the electron coupling term, f_K is the Fermi distribution of the electrons, ε is the state energy for hopping, and $F(\Delta E)$ is the thermally averaged Franck-Condon term that accounts for the the nuclear and environment relaxation which is given by:

$$F(\Delta E) = \frac{1}{2\sqrt{\pi \lambda k_b T}} \exp\left(-\frac{(\Delta E - \lambda)^2}{4\lambda k_b T}\right) \quad (7)$$

where λ is the reorganization energy and ΔE is the energy change of the system upon charge hopping. The current in the hopping model can be expressed as:

$$I^h = q \cdot \frac{R_{AB}^{L} R_{BA}^{R} - R_{AB}^{R} R_{BA}^{L}}{R_{AB} + R_{AB}} \quad (8)$$

Equation 8 was used to plot and compare experimental data to the hopping model I-V response.

Based on Eq. 4, the tunneling current at low bias increases nearly linearly with voltage. In contrast, based on Eq. 8, the hopping current exhibits a non-linear (exponential) relationship with increasing voltage, which results in the I-V hopping curve spanning a significantly larger range of current across a similar range of applied bias compared to tunneling-dominated transport.[2] As shown in **Figure S18a**, our analysis shows that the hopping transport model provides good agreement with the I-V behavior of peptide monolayers with heme. In contrast, the tunneling model shows a markedly different behavior and fails to provide agreement with the I-V behavior of peptide monolayers with heme regardless of parameter selection. On the other hand, the

tunneling model shows good agreement with peptide monolayer I-V behavior in the absence of heme, as shown in **Figure S18b**. These results suggest that without heme, charge transport mainly occurs by tunneling, and the addition of heme introduces hopping sites that greatly enhance electron transport in these materials.

The parameters of these models were chosen to fit the experimentally observed current density at an applied bias of -1 V. The key fitting parameters in the hopping model include the reorganization energy $\lambda = 0.5$ eV, the initial energy difference relative to electrode Fermi energy $E - \varepsilon = 0.1$ eV, and temperature $T = 298$ K. These values are in good agreement with previously reported literature values for heme redox reorganization energy (0.45 eV - 0.48 eV)[4] and a small energy difference between heme HOMO level energy (close to -5 eV)[5] and Au Fermi energy (~ -5 eV relative to vacuum level). The effect of the electronic coupling parameter k is to primarily shift the I-V curve across different orders of magnitude without altering the qualititative response or I-V curve shape. In our measurements, which involve a large contact area where the current density is proportional to the contact area, our focus is on capturing the overall behavior and shape of the I-V curve rather than the precise quantitative values. Consequently, as discussed in prior work, attempting to fit curves to capture the absolute values is not meaningful in this context.[2]

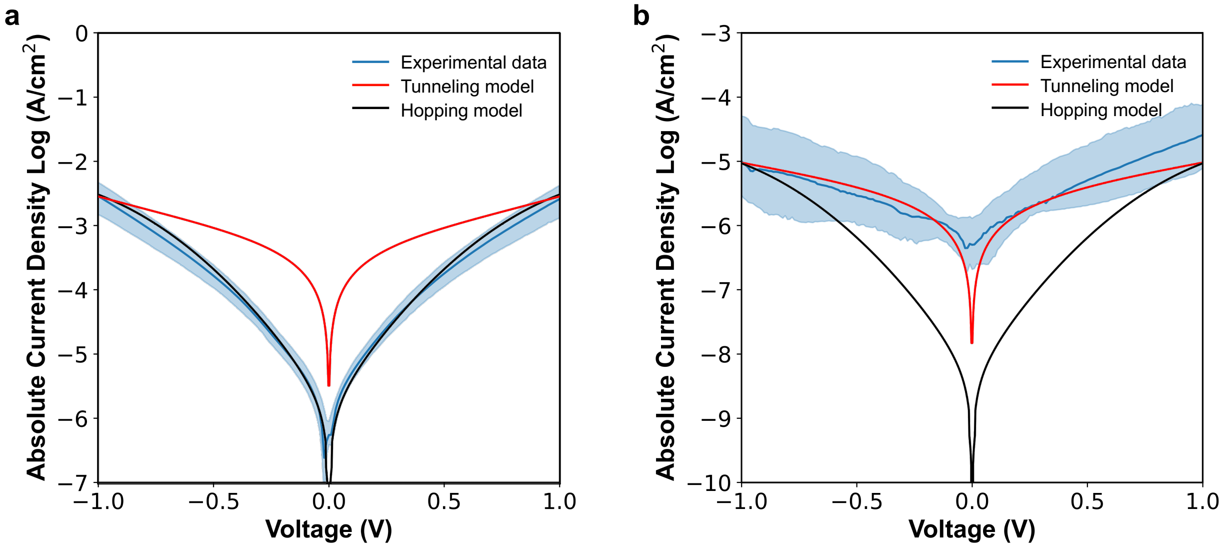

Figure S18. Current-voltage (I-V) plots of experimental data for (a) peptide sequence PAH at a heme:peptide ratio of 8:1 and (b) peptide sequence PAA without heme, compared to the I-V response predicted from the hopping model and tunneling model.

8 Molecular Dynamics Simulations

8.1 Modeling an initial monolayer structure with rigid-body packing

Molecular dynamics simulations were carried out to model monolayer assembly at varying packing densities. The initial structure of the PHH monomer was predicted using ESMFold, resulting in an amphiphilic helix decorated with nonpolar sidechains on one face and polar sidechains on its opposite face (**Figure S19**), in good agreement with the original heme-binding maquette design.[6] The PHH monomer's secondary structure mostly consists of a single contiguous α-helix which is connected to its N-terminal polyproline repeat by a turn, as shown in **Figure S20**. The ESMFold-predicted monomer structure was then used in the following steps of the procedure to model assembled monolayer systems.

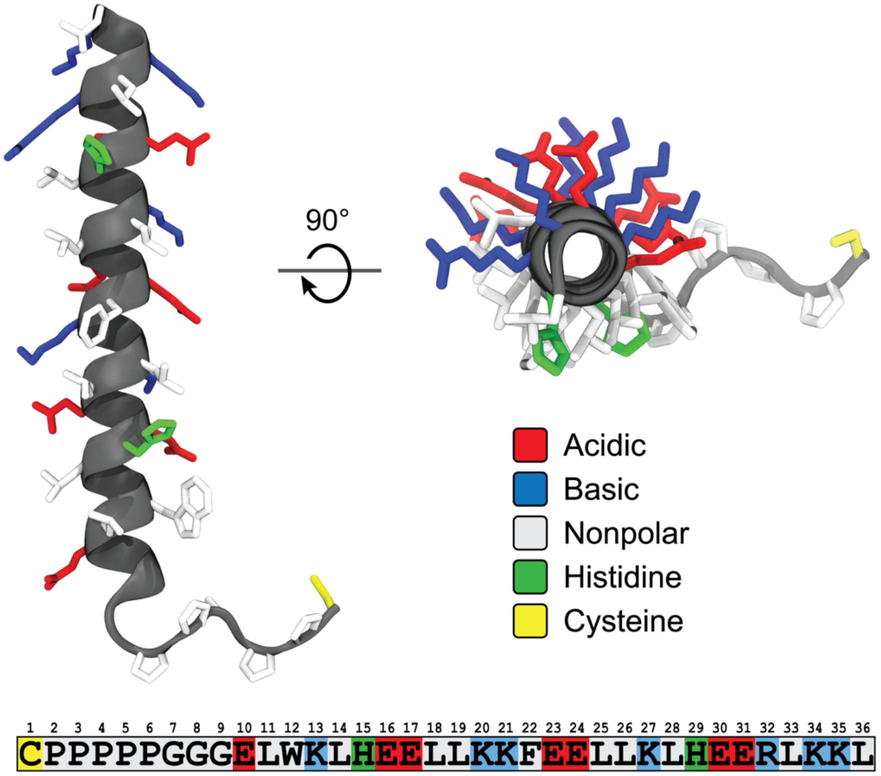

Figure S19. Amphiphilic PHH peptide monomer structure predicted by ESMFold, with side view (left) and top view (right). Peptide backbone is shown in cartoon representation (dark gray) and sidechains are shown in licorice representation (multicolor). The input sequence (bottom) is colored by residue type (acidic, basic, and nonpolar). Heme-binding (histidine) and gold-binding (cysteine) sidechains are colored separately. Images were rendered using VMD.[7]

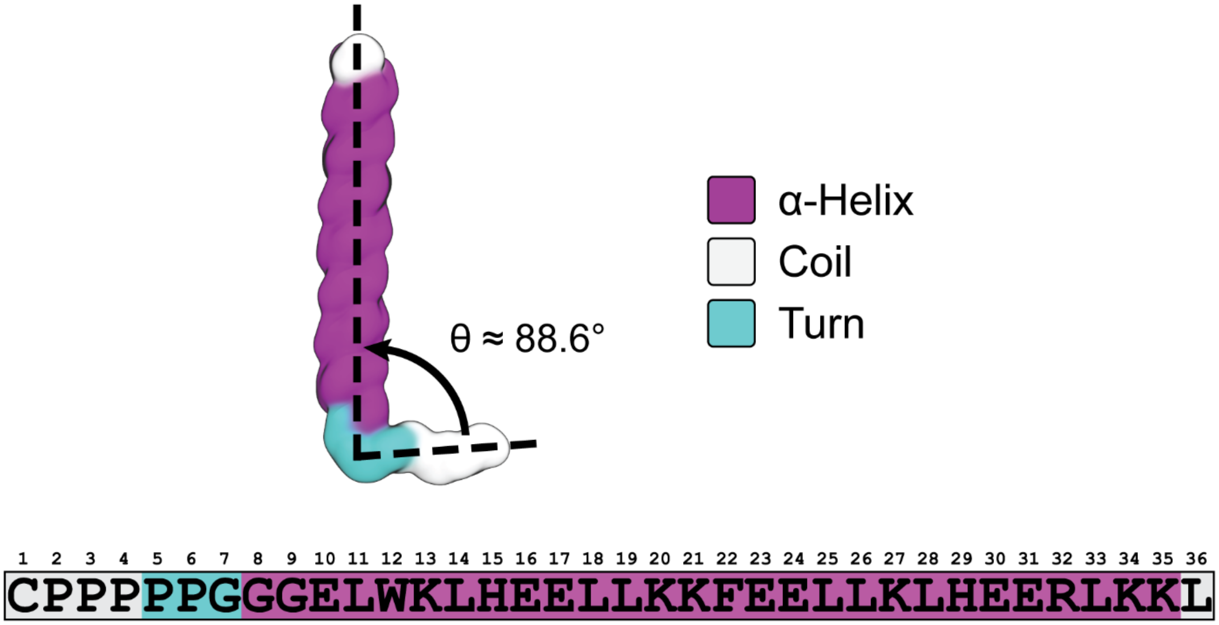

Figure S20. PHH peptide monomer secondary structure predicted by ESMFold. Secondary structure assignment is performed using STRIDE[8] as implemented in VMD [7] the depicted angle θ is calculated as the angle between the first principal axes of the polyproline (residues 1–6) and α-helix (residues 8–35) alpha carbons.

Initial monolayer structures were modeled with spatially constrained rigid-body packing using Packmol[9] (**Figure S21**). A range of packing densities were modeled by varying the number of peptide chains to be packed in the same 10 nm x 10 nm area. The positions of the backbone nitrogen atoms of the first six N-terminal residues were constrained below the plane at z = 1 nm and the position of the C-terminal residue's backbone nitrogen atom was constrained above the plane at z = 4 nm. These values were chosen to ensure the monolayer's height is initialized close to the experimentally measured height of approximately 3 nm. For each of the five packing density conditions, three replicate monolayers were independently generated with different random seeds using this approach. The initial structures of each monolayer replicate is shown in **Figure S22**.

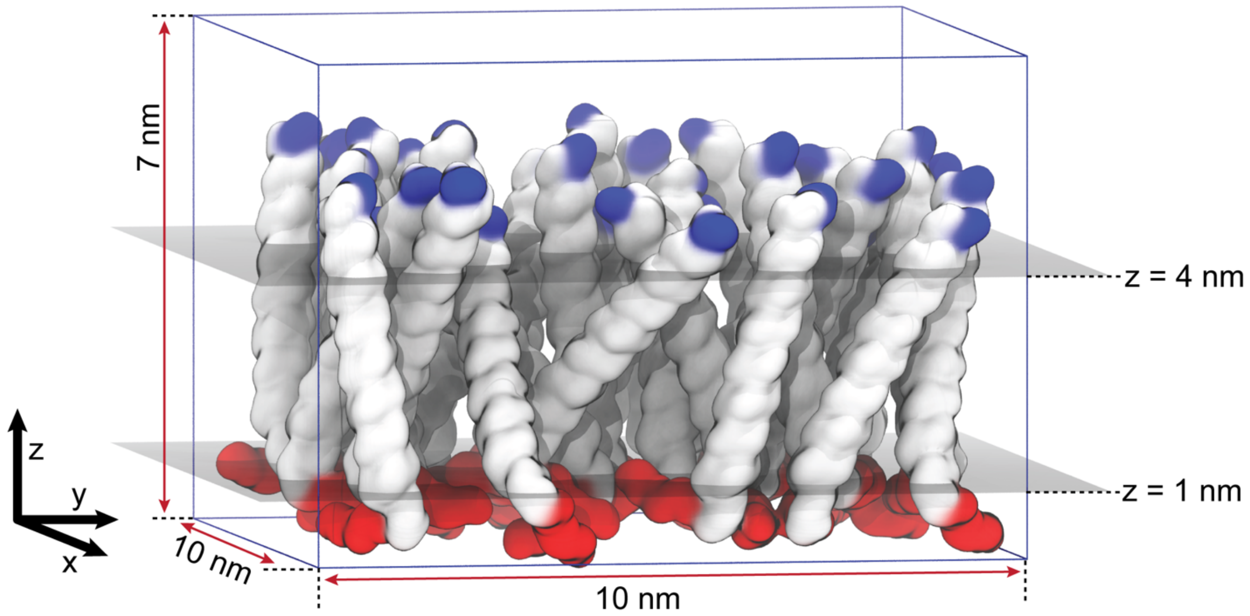

Figure S21. Schematic depiction of the rigid-body packing strategy used to generate initial monolayer structures, showing one replica of the 30-helix packing condition. Using Packmol, peptide chains are packed in a 10 nm × 10 nm area. Packing is constrained such that the backbone nitrogen atoms of the residues depicted in red (residues 1–6) must reside below the plane at z = 1 nm, while the backbone nitrogen of the C-terminus (residue 36) must reside above the plane at z = 4 nm. The portion of peptide shown in white is not subject to spatial packing constraints. The unit cell of the solvated system is depicted as a blue box, with unit cell lengths shown by red arrows. Image rendered using VMD.[7]

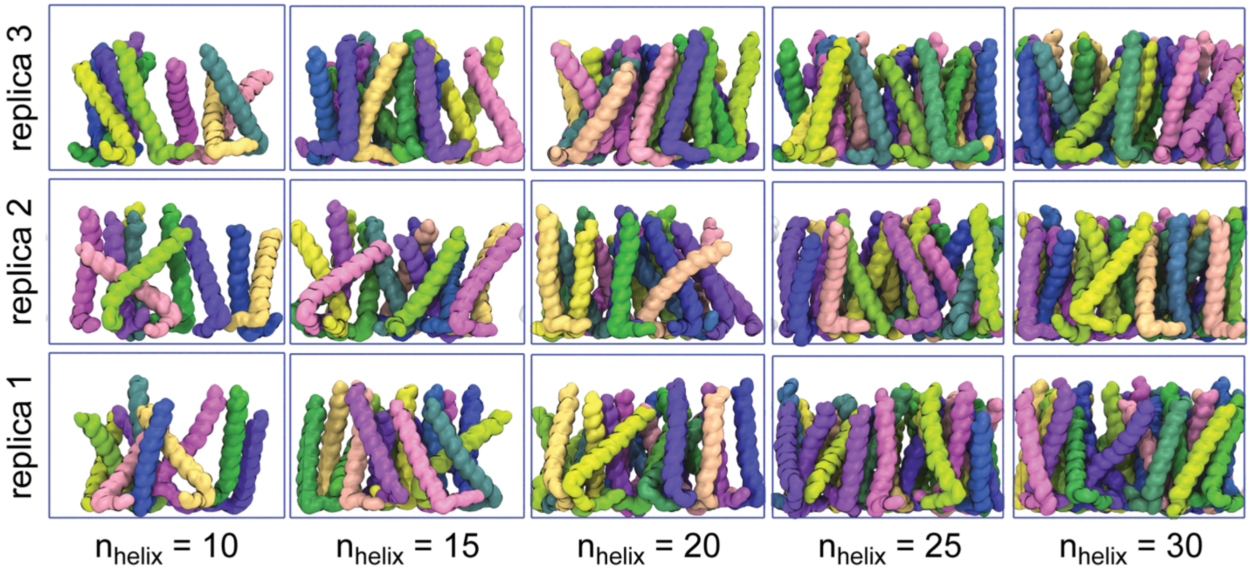

Figure S22. Initial monolayer structures prepared with rigid-body packing. Five packing conditions are modeled in triplicate. Peptide helices are shown as surface representations of their backbone atoms; sidechains are hidden for clarity.

8.2 MD simulations of monolayer assembly

After initial packing assemblies were generated, each replicate was subjected to molecular dynamics simulation with a set of custom restraining potentials to implicitly model its interactions with a planar gold substrate. The positions of all cysteine sulfur atoms were harmonically restrained to the plane at $z = 1$ nm with force constant $k = 418.4$ kJ/(mol nm^2) to mimic a covalent bond between each helix and the implicit substrate, following Equation 9:

$$U_S = \sum_{i=1}^{n_{helix}} \frac{1}{2} k_s \left(z_{S_i} - z_0\right)^2 \tag{9}$$

where z_{S_i} is the z-coordinate of the sulfur atom of helix i, the equilibrium position of the harmonic restraint is z_0, and the value of the force constant k_S is 418.4 kJ/(mol nm^{-2}). Though the z-position of each helix's sulfur atom is restrained in the z-direction, each helix chain is free to diffuse within the x-y plane to allow the initially randomly packed helices to find and make favorable and stable contacts. To prevent protein diffusion into the implicit substrate, a repulsive potential was placed at the bottom of the simulation box such that any helix that diffuses below the plane at $z = 0.5$ nm is subjected to a constant force in the $+z$ direction. This excluded volume potential is subjected to all protein heavy atoms with a softplus functional form, which smoothly switches between zero force and constant force near the defined switching distance as defined in Equation 10:

$$U_{excl.vol.} = \sum_{i=1}^{n_{prot}} \frac{k_{excl.vol}}{b} ln\left(1 + \exp\left[b\left(z_{prot} - z_{switch}\right)\right]\right) \tag{10}$$

where the force constant $k_{excl.vol}$ is –10 kJ/(mol nm), n_{prot} is the number of protein heavy atoms in the system, the switching parameter b which controls the curvature of the switching potential is –50 nm^{-1}, and z_{switch} is 0.5 nm. This softplus potential is designed such that its derivative with respect to position (the force) has the form of a logistic function, resulting in zero force being applied to protein atoms in the region of the box above the switching plane, while a constant force of 10 kJ/(mol nm) repels any protein heavy atom that diffuses into the excluded volume region, with the force being smoothly switched on near the switching plane. Each applied potential is visualized in **Figure S23b**. Peptide N- and C-termini were modeled as charged using NTER and CTER patches, respectively. Each system was then solvated in an orthorhombic box of TIP3P water with x-dimension of 10 nm, y-dimension of 10 nm, and a z-dimension (height) of 7 nm using VMD Solvate.[7]

Molecular dynamics simulations were carried out using OpenMM 7.7.0[10] and the CHARMM36m force field.[11,12] Restraints were implemented using OpenMM's custom force classes. Each replicate was simulated for 2 μs, resulting in 30 μs of simulation in aggregate (5 packing conditions x 3 replicates). Dynamics were integrated using the Langev-inMiddleIntegrator[13] with friction coefficient of 1 ps^{-1}, temperature of 300 K, and a timestep of 2 fs. Simulation pressure was controlled at 1 atm using a Monte Carlo barostat with frequency of 25 timesteps. Atomic coordinates were written to disk every 10 ps. Bonds involving hydrogen atoms, and all bonds and angles involving water were constrained. Nonbonded interactions were computed with a cutoff of 1.2 nm with smooth switching starting at 1.0 nm. Electrostatic interactions were evaluated using particle mesh Ewald[14] (PME) summation with error tolerance of 0.0005.

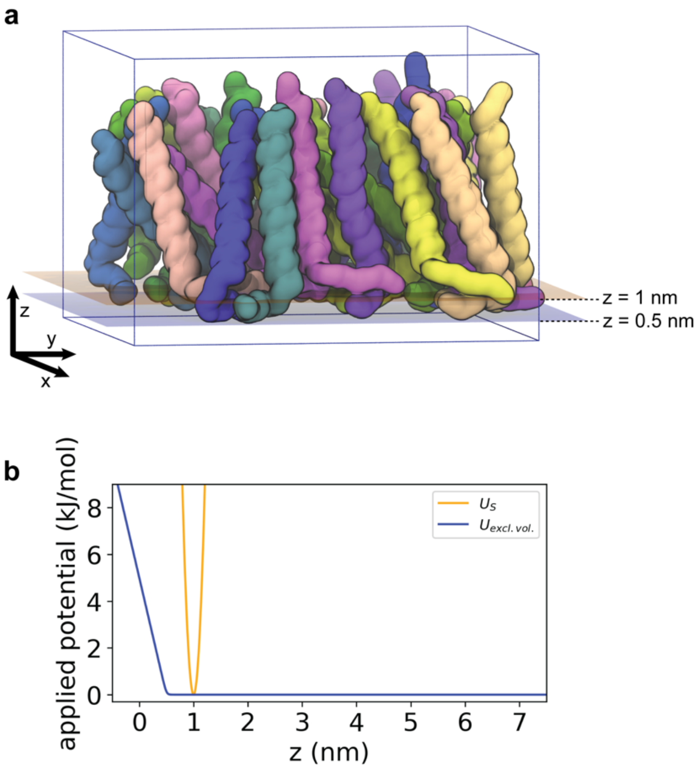

Figure S23. Schematic of external potentials applied during MD simulation. (a) 30-helix system during assembly with applied potentials. Anchor thiol sulfur atoms are restrained to the plane at z = 1 nm (orange transparent plane) while all protein heavy atoms are subjected to a repulsive potential starting at z = 0.5 nm (blue transparent plane). The periodic unit cell of the system is depicted as a blue box. (b) Energy profiles of applied potentials. The sulfur-restraining potential as defined in Equation 9 is shown in orange, while the excluded-volume potential as defined in Equation 10 is shown in blue.

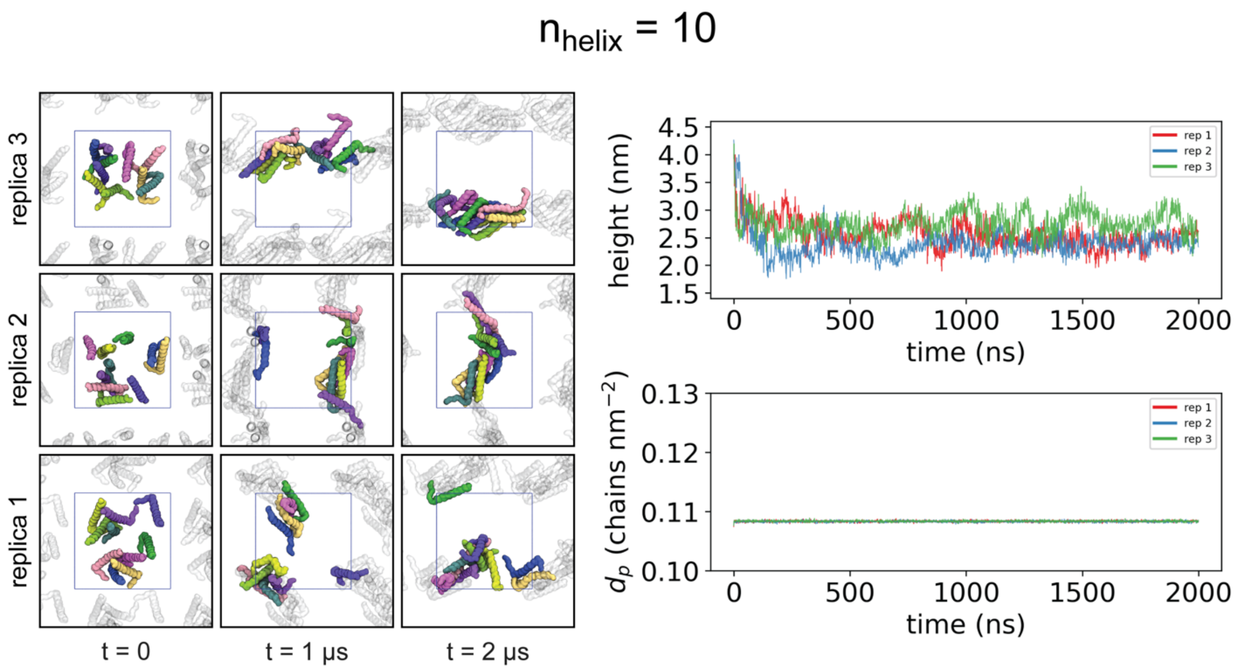

Figure S24. MD simulations of 10-helix monomer assembly. Top-view snapshots for each replica are shown at the beginning, middle, and end of each simulation (left). For each system snapshot, the periodic unit cell is depicted as a blue box while peptide chains are shown as multicolored backbone surfaces. Periodic images in the *x*- and *y*-directions are shown as grey transparent surfaces. The height (top right) and packing density (bottom right) of each replicate is plotted versus time.

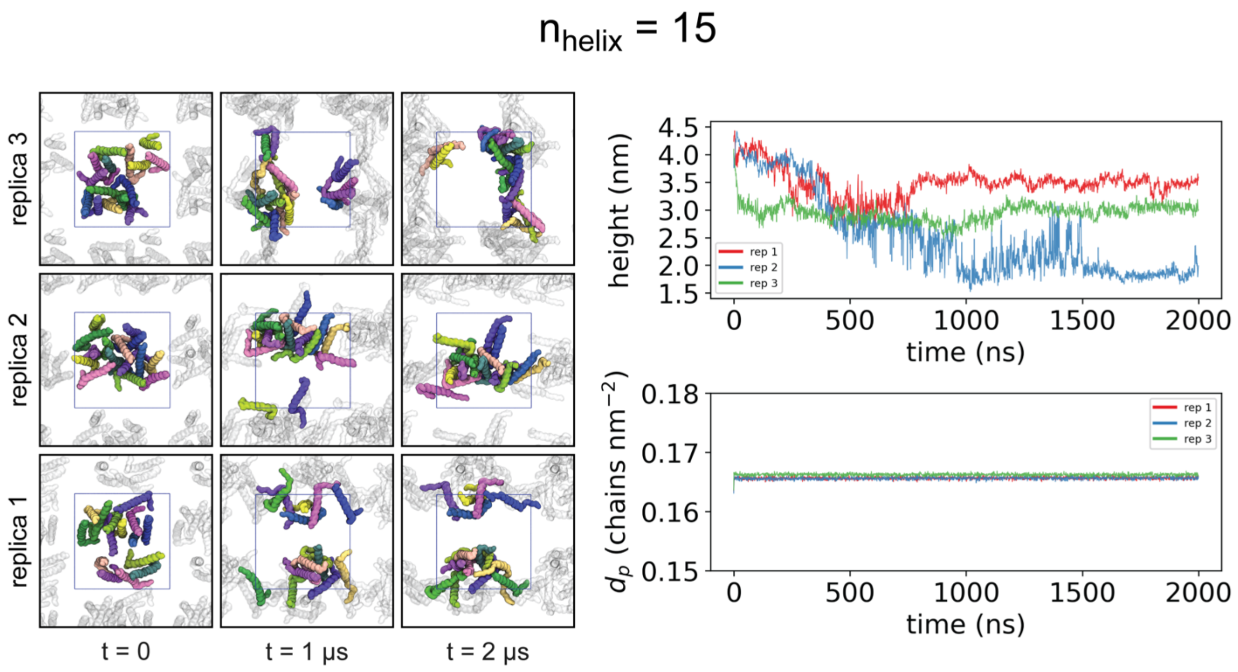

Figure S25. MD simulations of 15-helix monomer assembly. Top-view snapshots for each replica are shown at the beginning, middle, and end of each simulation (left). For each system snapshot, the periodic unit cell is depicted as a blue box while peptide chains are shown as multicolored backbone surfaces. Periodic images in the *x*- and *y*-directions are shown as grey transparent surfaces. The height (top right) and packing density (bottom right) of each replicate is plotted versus time.

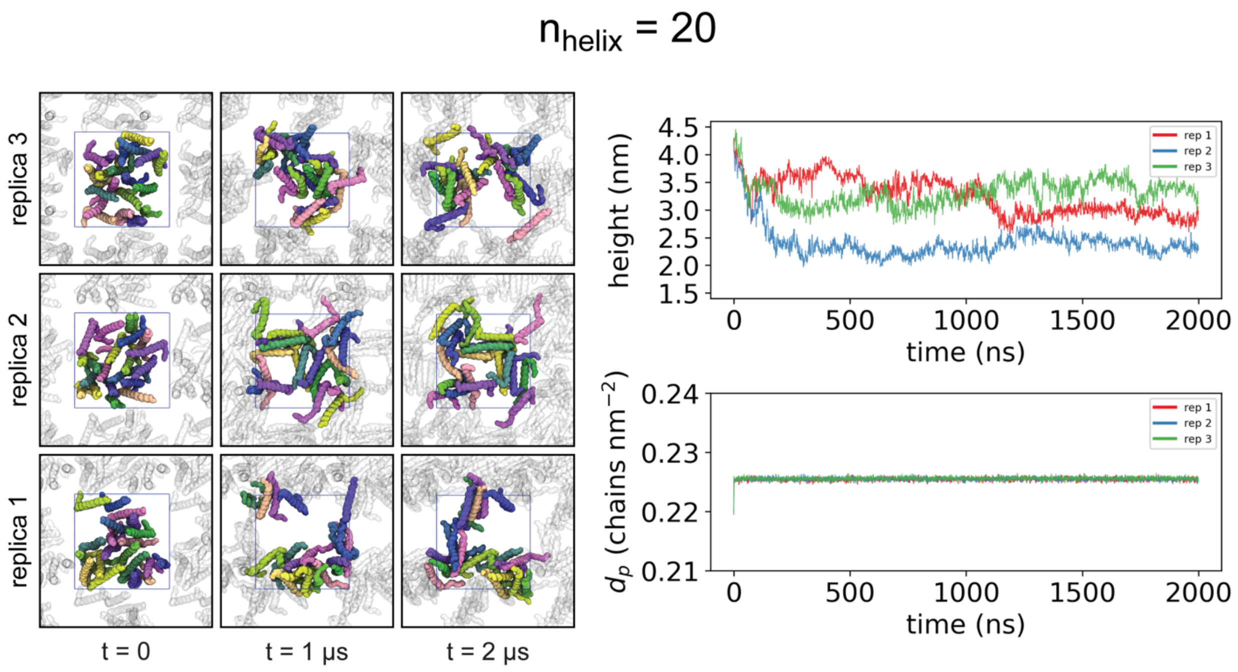

Figure S26. MD simulations of 20-helix monomer assembly. Top-view snapshots for each replica are shown at the beginning, middle, and end of each simulation (left). For each system snapshot, the periodic unit cell is depicted as a blue box while peptide chains are shown as multicolored backbone surfaces. Periodic images in the *x*- and *y*-directions are shown as grey transparent surfaces. The height (top right) and packing density (bottom right) of each replicate is plotted versus time.

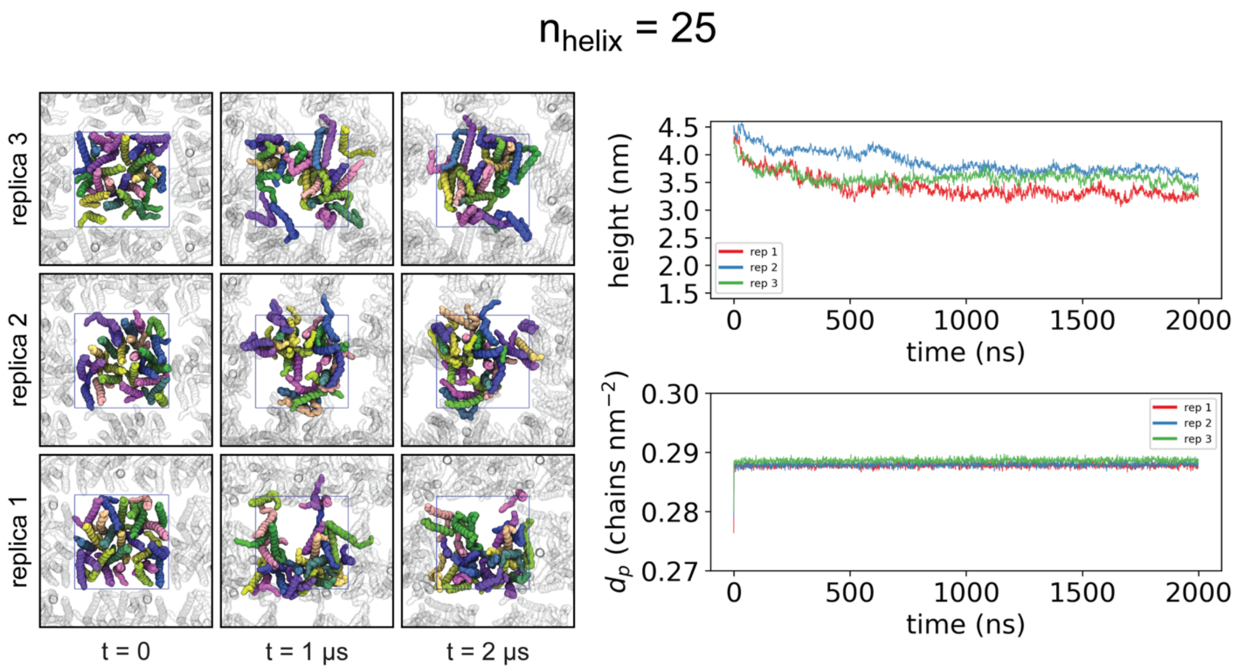

Figure S27. MD simulations of 25-helix monomer assembly. Top-view snapshots for each replica are shown at the beginning, middle, and end of each simulation (left). For each system snapshot, the periodic unit cell is depicted as a blue box while peptide chains are shown as multicolored backbone surfaces. Periodic images in the x- and y-directions are shown as grey transparent surfaces. The height (top right) and packing density (bottom right) of each replicate is plotted versus time.

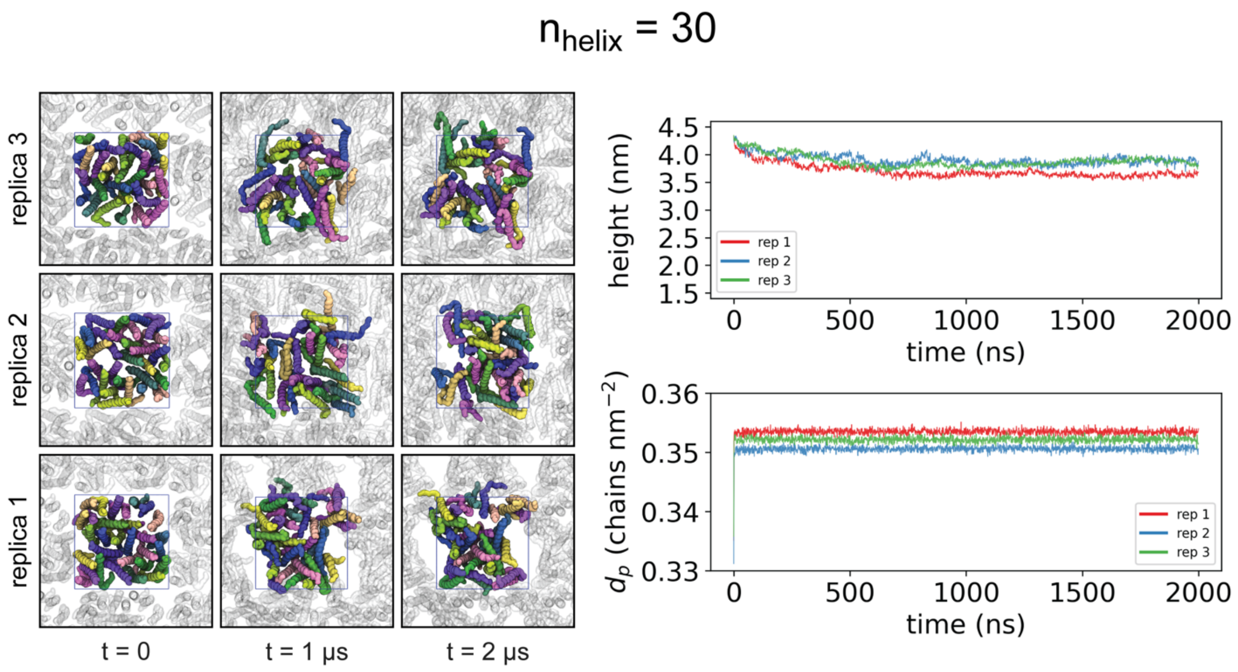

Figure S28. MD simulations of 30-helix monomer assembly. Top-view snapshots for each replica are shown at the beginning, middle, and end of each simulation (left). For each system snapshot, the periodic unit cell is depicted as a blue box while peptide chains are shown as multicolored backbone surfaces. Periodic images in the *x*- and *y*-directions are shown as grey transparent surfaces. The height (top right) and packing density (bottom right) of each replicate is plotted versus time.

8.3 Calculating monolayer height and packing density

To investigate the relationship between packing density and height, these properties were calculated for each monolayer system every 1 ns (100 simulation frames) over the 2 μs trajectory. The packing density d_p of each monolayer system was calculated as the number of helix chains divided by the product of the unit cell lengths in the *x*- and *y*-dimensions, as shown in Equation 11:

$$d_p = \frac{n_{helix}}{a \bullet b} \tag{11}$$

where n_{helix} is the number of helix chains, and *a* and *b* are the PBC unit cell lengths in the *x*- and *y*-dimensions. Next, the height of each peptide monolayer was calculated in order to compare with experimental (AFM) height measurements. Monolayer height calculations from MD coordinates are treated as a problem of boundary detection: we define the height of the monolayer as the difference between the positions of its upper and lower boundaries. Thus, the boundaries of the monolayer should be calculated in a way that is robust to small local deviations in height. To this end, we define the boundaries of the peptide monolayer as the upper and lower half-max of the protein heavy atom density along the monolayer normal (*z*) axis, resulting in the height being calculated as the full width at half-max.

$$h = FWHM(\{z_{prot,i}\}_{i=1\ldots n_{prot}}) \tag{12}$$

where $z_{prot,i}$ is the *z*-coordinate of protein heavy atom *i*, and n_{prot} is the number of protein heavy atoms.

8.4 Weighted least-squares regression

Three replicate simulations were performed at each of the five packing density conditions in this study (10, 15, 20, 25, or 30 chains per 100 nm^2), resulting in 15 independent monolayer simulations. For each system, the height and packing density are calculated every 1 ns (equivalent to stride of 100 frames). The means $\{\bar{h}_i\}_{\mathrm{i}=1\ldots15}$ and variances $\{\sigma^2(h_i)\}_{\mathrm{i}=1\ldots15}$ of the height in the final 500 ns of each simulation is then calculated. We calculate packing density (d_p) every 1 ns and record the mean d_p values of each system over the last 500 ns of simulations as $\{\bar{d}_{pi}\}_{\mathrm{i}=1\ldots15}$. The mean values $\bar{d}_{pi}$ and $\bar{h}_i$ and the variance of the height $\sigma^2(h_i)$ are provided to a weighted least-squares (WLS) regression model to calculate a regression line of height versus packing density that assigns a

higher penalty to residuals of high-certainty data and lower penalty to residuals of low-certainty data, as shown below:

$$\beta = (X^\top W X)^{-1} X^\top W_y \tag{13}$$

where β is the 2×1 vector containing the slope and intercept of the WLS regression line, X is the 15×2 design matrix with packing density values $\bar{d}_{pi}$ of each system in the first column and ones in the second column, W is the 15×15 diagonal weight matrix with each diagonal element W_{ii} being equal to the inverse of the variance of each system's height $1/\sigma^2{}_{h_i}$, and y is the 15×1 vector containing each system's height $\bar{h}_i$. WLS parameter variances are calculated as the diagonal elements of the matrix inverse of $(X^\top W X)^{-1}$.

8.5 Simulating heme infiltration into peptide monolayer

A series of molecular dynamics simulations were carried out to model heme diffusion into heme-binding helical monolayers. First, a monolayer consisting of 30 PHH helix chains was prepared and equilibrated with restraints following the procedures detailed in subsections 7.1 and 7.2 respectively, and the structure of the solvated monolayer was extracted at the last frame of equilibration. The resulting PHH monolayer structure was then used to generate structures of PAH, PHA, and PAA monolayers by performing alanine point mutations at the position of the first, second, or both histidine residues, resulting in a total of four monolayer systems with varying sequence (PHH, PAH, PHA, PAA) and near-identical assembly. Point mutants were generated using the mutate command in the psfgen plugin of VMD,[7] followed by a local energy minimization with constraints on the unmutated residues. Minimization constraints in OpenMM were applied by setting the mass of constrained particles to zero during minimization. A heme solution was added to each of the four systems. First, a slab of heme solution was prepared using Packmol[9] by packing 30 heme molecules into a slab with *x*- and *y*-dimensions matching each monolayer (≈9.4 nm) and *z*-dimension of 1 nm. The heme molecules were then solvated with TIP3P water and ionized with sodium counterions to neutral charge. The resulting slab of heme solution was placed directly above the equilibrated monolayer. This procedure was repeated three times for each sequence yielding three independent replicas for each sequence, with the placement of heme being randomized for each replica.The combined systems were then equilibrated for 20 ps with a series of custom restraints, detailed below.

The positions of all cysteine sulfur atoms were restrained to the plane at z = 1 nm following Equation 9. The positions of protein and heme heavy atoms were also restrained above the implicit substrate's excluded volume following Equation 10 with force constant $k_{excl.vol.}$ of –41.84 kJ/(mol nm).

Periodic boundary conditions are being utilized in our MD simulations, so we must ensure that heme diffusion into the monolayer proceeds from the solution volume above the peptide monolayer. To this end, another softplus repulsive potential was added (following Equation 10) with parameters b of 50 nm^{-1}, force constant $k_{excl.vol.}$ of 41.84 kJ/(mol nm), and switching plane position zswitch at 7.0 nm, resulting in a constant force being applied to any heme heavy atoms that diffuse above the plane at z = 7 nm (**Figure S29**).

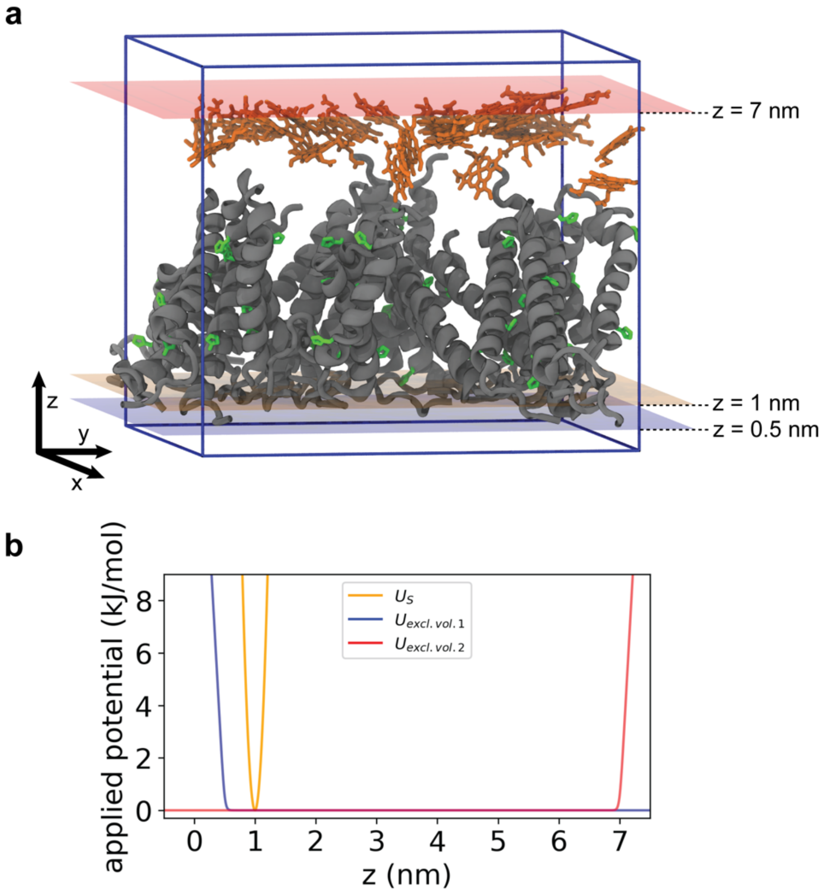

Figure S29. Schematic of external potentials applied during MD simulation of heme permeation. (a) 30-helix system during heme permeation simulation with applied potentials. Anchor thiol sulfur atoms are restrained to the plane at z = 1 nm (orange transparent plane) while all protein and heme heavy atoms are subjected to a repulsive potential starting at z = 0.5 nm (blue transparent plane).

All heme heavy atoms are also subjected to a repulsive potential starting at z = 7 nm. The periodic unit cell of the system is depicted as a blue box. (b) Energy profiles of applied potentials. The sulfur-restraining potential as defined in 9 is shown in orange, while two excluded-volume potentials as defined in Equation 10 are shown in blue and red.

Finally, for all monolayer systems containing histidine (PHH, PAH, PHA), an attractive pairwise potential between every heme iron atom and every histidine *ε*-nitrogen atom was added in the form of a Morse potential, as defined in Equation 14:

$$U_{Morse} = \sum_{i=1}^{n_{heme}} \sum_{j=1}^{n_{his}} E_0 \left(1 - \exp\left[-a\left(r_{ij} - r_0\right)\right]\right)^2 \tag{14}$$

where n_{heme} is 30, $n_{his}$is the number of histidine residues, and r_{ij} is the distance between the iron atom of heme molecule *i* and the *ε*-nitrogen atom of histidine residue j. Morse potential parameters E_0, r_0, and a are respectively set to 87.0 kJ/mol, 0.205 nm, and 30.3 nm^{-1} following previous QM calculations of iron-porphyrin–imidazole dissociation in the literature.[15]

We also implement an anti-aggregation potential for heme by modifying the energy associated with heme-heme nonbonded interactions using NBFix parameters. The Lennard-Jones parameters for the interaction energy between heme molecules' π-conjugated heavy atoms was adjusted such that the depth of the energy well ϵ_{ij} is zero.

The atom types for which ϵ_{ij} is set to zero are CPA, CPB, CPM, and NPH (**Figure S30b**), using atom type names as defined in CHARMM36 stream parameter files.[16] This results in LJ forces between stacking moieties on heme molecules always being zero, which nearly completely removes the tendency for π-stacking interactions between heme molecules without compromising heme-protein and heme-solvent interactions. Control simulations for heme solutions with and without the NBFix anti-aggregation potential show a marked decrease in aggregation with NBFix, illustrated by the change in Fe–Fe radial distribution functions for each condition (**Figure S30**). The control system was created by randomly packing 10 heme molecules into a 5 nm × 5 nm × 5 nm box with Packmol, followed by addition of water and counterions. The heme solution was simulated for 10 ns with and without NBFix parameters.

Using the above defined potentials, MD simulations of each replica of each monolayer-heme system were carried out for 4 μs, resulting in 48 μs of total sampling (3 replicas × 4 sequences × 4

μs). Heme binding to histidine was calculated as the number of heme molecules with their iron atom within 0.25 nm of a histidine residue's ε-nitrogen. Nonspecific heme binding was calculated using the coordNum between all heme heavy atoms and all protein heavy atoms as follows:

$$coordNum = \sum_{i=1}^{n_{heme}} \sum_{j=1}^{n_{prot}} \frac{1-(\frac{r_{ij}}{r_0})^6}{1-(\frac{r_{ij}}{r_0})^{12}} \tag{15}$$

where n_{heme} is the number of heme heavy atoms, n_{prot} is the number of protein heavy atoms, r_{ij} is the distance between heme atom i and protein atom j, and r_0 is 0.3 nm.

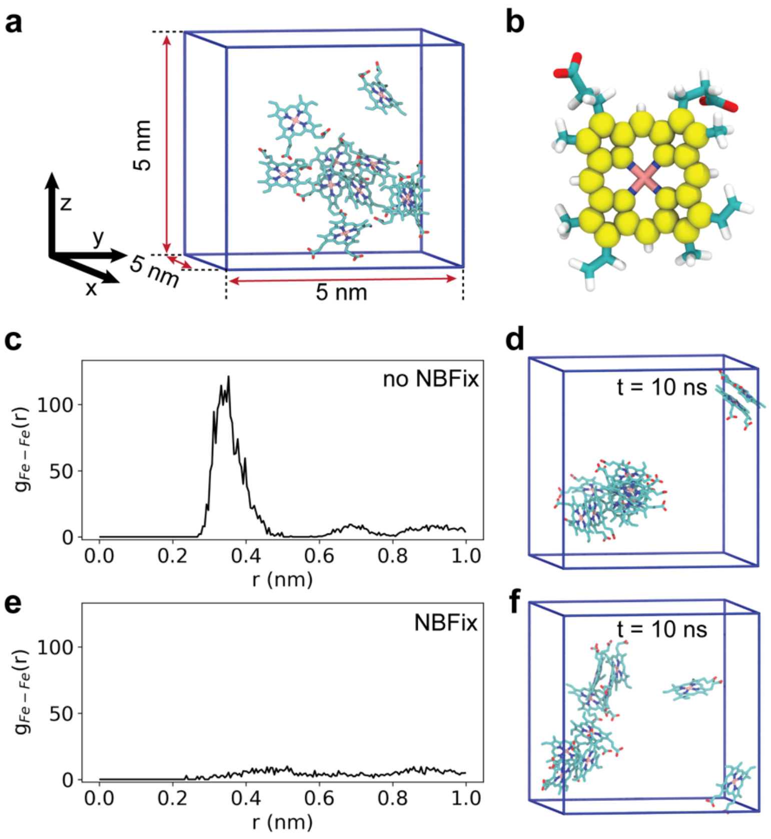

Figure S30. Control simulations for reducing heme aggregation in solution. (a) Initial setup of 10 heme molecules in an orthorhombic unit cell (blue box) with side length of 5 nm (red arrows). (b) A heme molecule. Yellow VDW spheres indicate the atoms for which anti-aggregation NBFix parameters were applied. (c) Radial distribution function between heme iron atoms computed over 10 ns simulation without NBFix anti-aggregation parameters. (d) System snapshot after 10 ns simulation without NBFix parameters, showing a high degree of π-stacking and aggregation. (e) Radial distribution function between heme iron atoms computed over 10 ns simulation with NBFix

anti-aggregation parameters. (f) System snapshot after 10 ns simulation with NBFix parameters, showing marked decrease in aggregation.

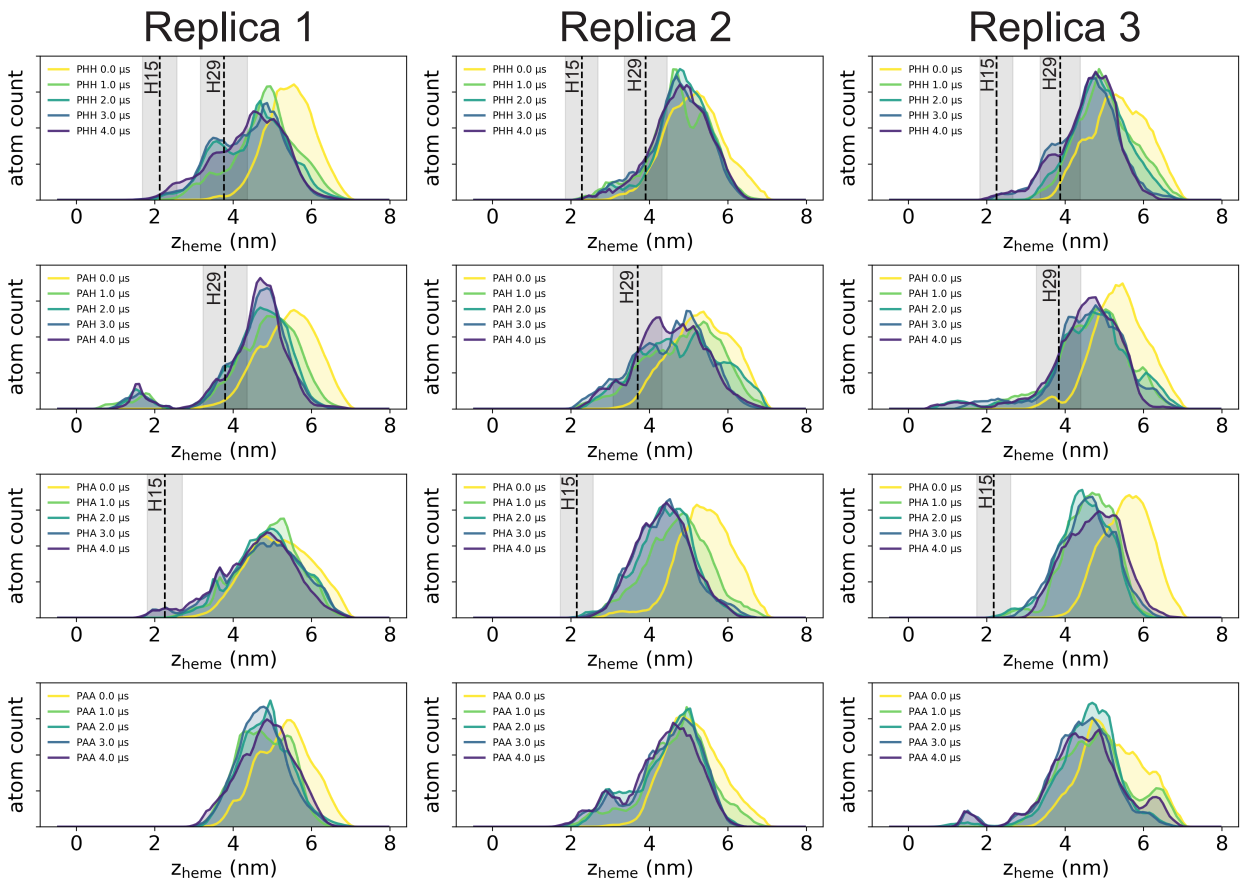

Figure S31. Permeation profile histograms showing heme heavy-atom position along monolayer normal axis over time. For histidine-containing monolayers, each histidine's average ± st.dev. z-position shown as vertical black dashed lines. PHH (top row), PAH (second row), PHA (third row) and PAA (last row) sequences are shown. Simulation replicas are indicated by column.

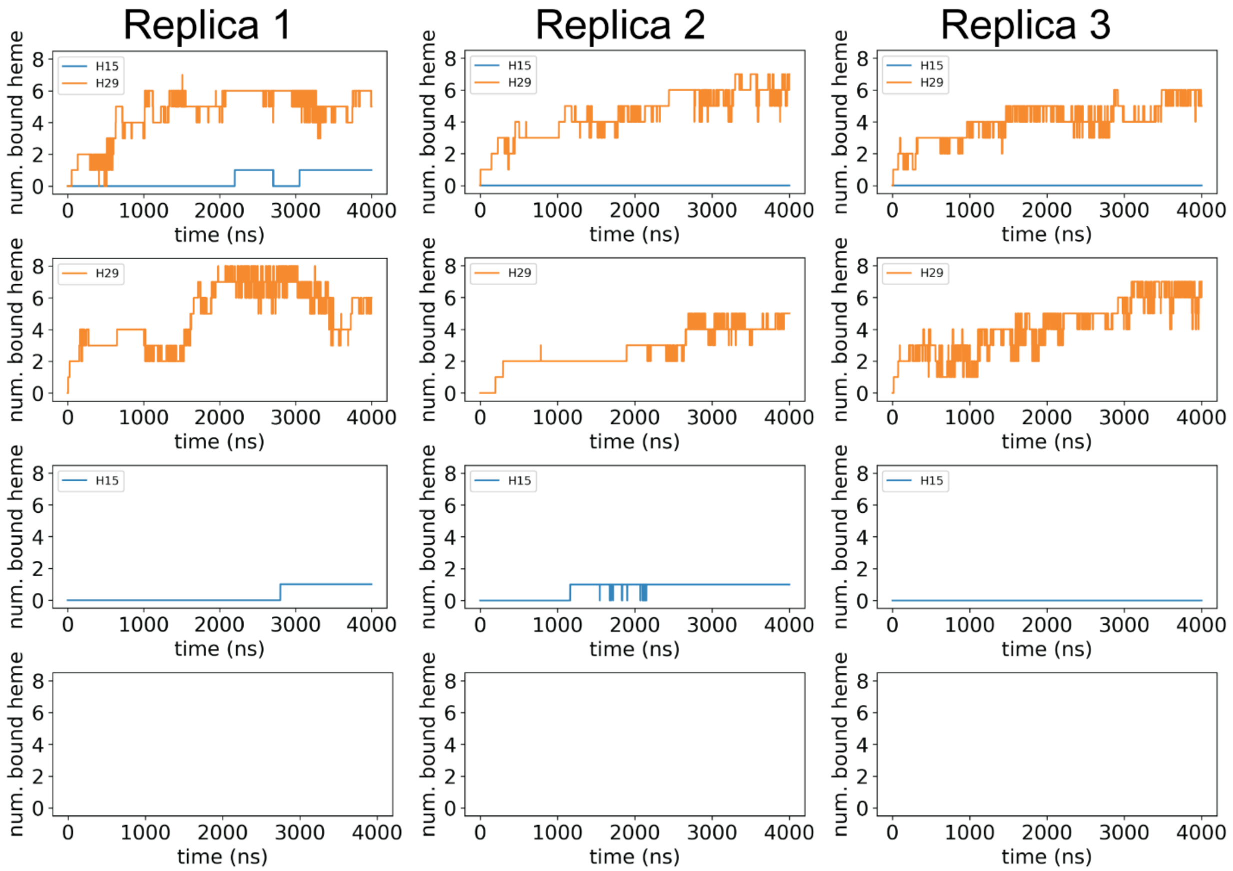

Figure S32. Number of histidine-bound heme molecules over time for each simulation replica of PHH (top row), PAH (second row), PHA (third row), and PAA (last row) peptide monolayers. No histidine-heme binding occurs in PAA monolayers due to the lack of histidine residues.

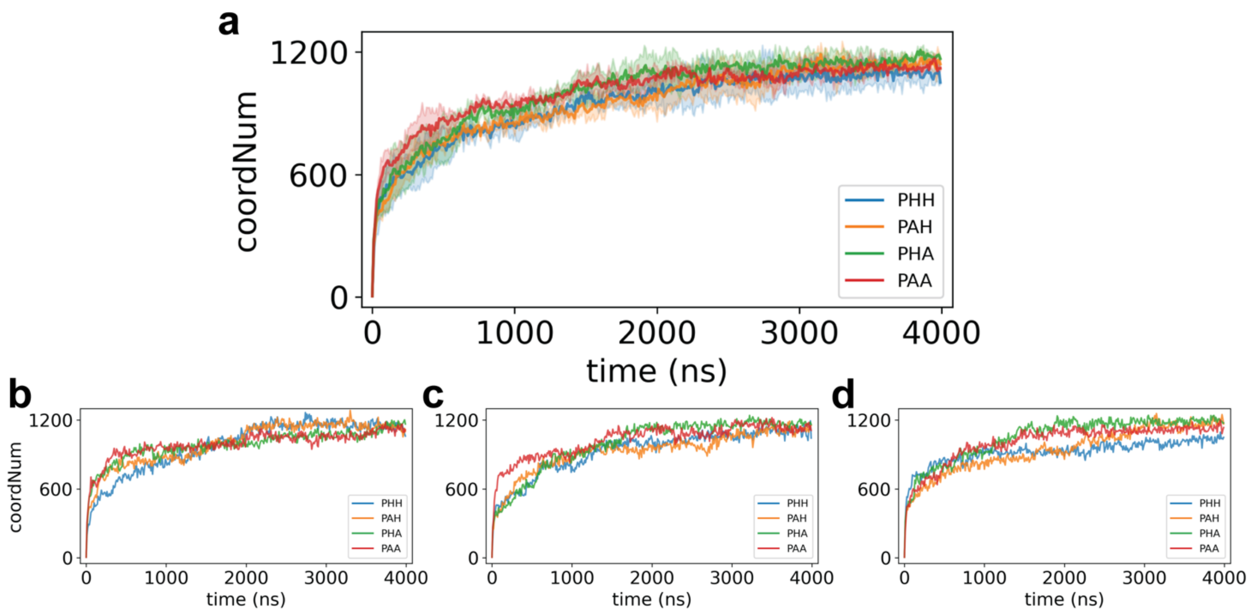

Figure S33. Timeseries analysis of nonspecific contacts between peptide monolayers and heme. Coordination number (coordNum; SI Equation 15) is calculated between all protein heavy atoms and all heme heavy atoms, with stride 10 ns, for each replicate of each peptide sequence. (a) Replica-averaged coordination number +/- standard deviation. (b) Coordination number timeseries plots for replica 1 (a), replica 2 (b), and replica 3 (c) are shown individually.

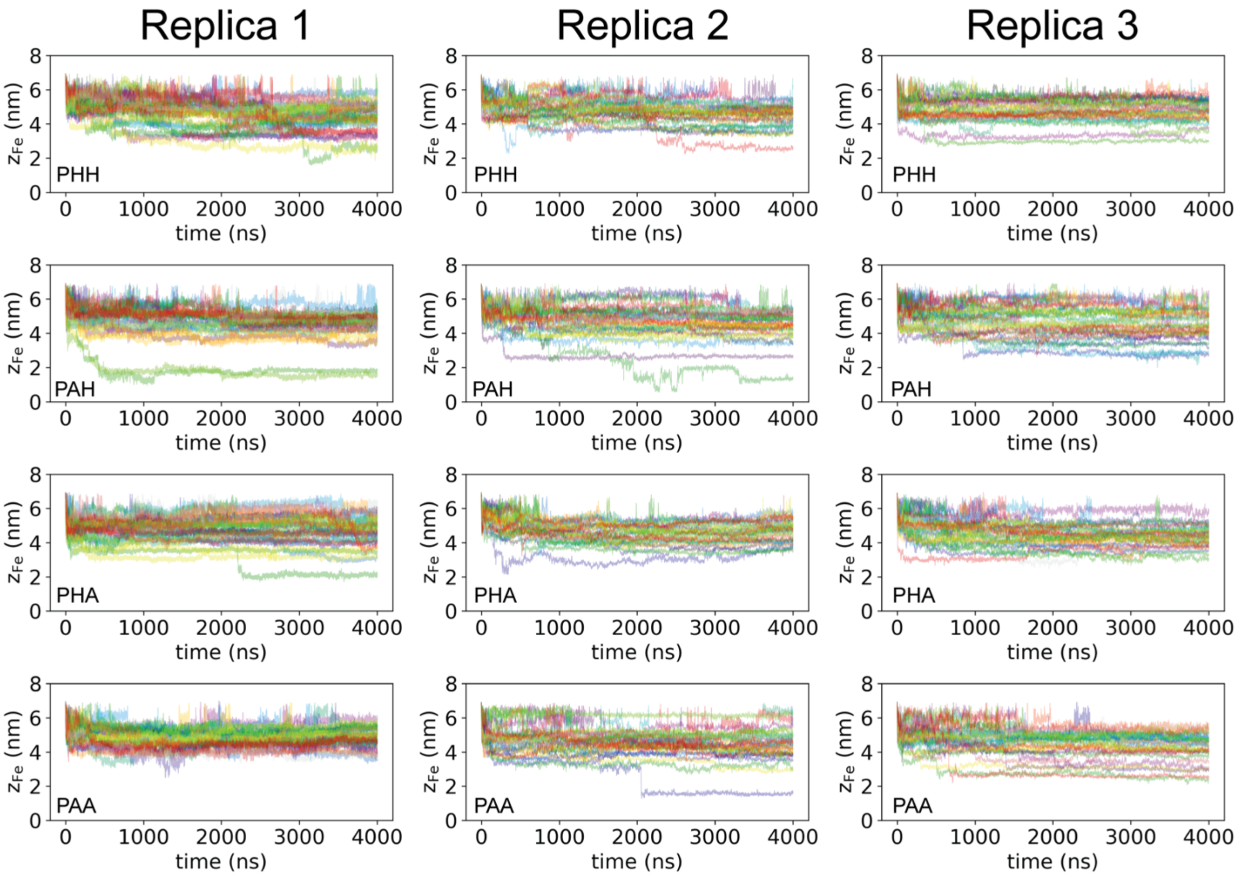

Figure S34. Position of individual heme molecules over time along the monolayer normal axis for sequences PHH (first row), PAH (second row), PHA (third row), and PAA (last row). Simulation replicas are indicated by column.

References

(1) Garrigues, A. R.; Yuan, L.; Wang, L.; Mucciolo, E. R.; Thompon, D.; del Barco, E.; Nijhuis, C. A. A Single-Level Tunnel Model to Account for Electrical Transport through Single Molecule- and Self-Assembled Monolayer-Based Junctions. *Sci Rep* **2016**, *6* (1), 26517.

(2) Song, X.; Han, B.; Yu, X.; Hu, W. The Analysis of Charge Transport Mechanism in Molecular Junctions Based on Current-Voltage Characteristics. *Chemical Physics* **2020**, *528*, 110514.

(3) Migliore, A.; Nitzan, A. Nonlinear Charge Transport in Redox Molecular Junctions: A Marcus Perspective. *ACS Nano* **2011**, *5* (8), 6669–6685.

(4) Krishtalik, L. I. The Medium Reorganization Energy for the Charge Transfer Reactions in Proteins. *Biochimica et Biophysica Acta (BBA) - Bioenergetics* **2011**, *1807* (11), 1444–1456.

(5) Ri, Y.-K.; Kim, S.-A.; Kye, Y.-H.; Jong, Y.-C.; Kang, M.-S.; Yu, C.-J. First-Principles Study of Molecular Hydrogen Binding to Heme in Competition with O 2 , NO and CO. *RSC Advances* **2024**, *14* (24), 16629–16638.

(6) Huang, S. S.; Gibney, B. R.; Stayrook, S. E.; Leslie Dutton, P.; Lewis, M. X-Ray Structure of a Maquette Scaffold. *Journal of Molecular Biology* **2003**, *326* (4), 1219–1225.

(7) Humphrey, W.; Dalke, A.; Schulten, K. VMD: Visual Molecular Dynamics. *Journal of Molecular Graphics* **1996**, *14* (1), 33–38.
(8) Frishman, D.; Argos, P. Knowledge-Based Protein Secondary Structure Assignment. *Proteins: Structure, Function, and Bioinformatics* **1995**, *23* (4), 566–579.
(9) Martínez, L.; Andrade, R.; Birgin, E. G.; Martínez, J. M. PACKMOL: A package for building initial configurations for molecular dynamics simulations. *Journal of Computational Chemistry* **2009**, *30* (13), 2157–2164.
(10) Eastman, P.; Swails, J.; Chodera, J. D.; McGibbon, R. T.; Zhao, Y.; Beauchamp, K. A.; Wang, L.-P.; Simmonett, A. C.; Harrigan, M. P.; Stern, C. D.; Wiewiora, R. P.; Brooks, B. R.; Pande, V. S. OpenMM 7: Rapid Development of High Performance Algorithms for Molecular Dynamics. *PLOS Computational Biology* **2017**, *13* (7), e1005659.
(11) Brooks, B. R.; Brooks III, C. L.; Mackerell Jr., A. D.; Nilsson, L.; Petrella, R. J.; Roux, B.; Won, Y.; Archontis, G.; Bartels, C.; Boresch, S.; Caflisch, A.; Caves, L.; Cui, Q.; Dinner, A. R.; Feig, M.; Fischer, S.; Gao, J.; Hodoscek, M.; Im, W.; Kuczera, K.; Lazaridis, T.; Ma, J.; Ovchinnikov, V.; Paci, E.; Pastor, R. W.; Post, C. B.; Pu, J. Z.; Schaefer, M.; Tidor, B.; Venable, R. M.; Woodcock, H. L.; Wu, X.; Yang, W.; York, D. M.; Karplus, M. CHARMM: The Biomolecular Simulation Program. *Journal of Computational Chemistry* **2009**, *30* (10), 1545–1614.
(12) Huang, J.; Rauscher, S.; Nawrocki, G.; Ran, T.; Feig, M.; de Groot, B. L.; Grubmüller, H.; MacKerell, A. D. CHARMM36m: An Improved Force Field for Folded and Intrinsically Disordered Proteins. *Nat Methods* **2017**, *14* (1), 71–73.
(13) Zhang, Z.; Liu, X.; Yan, K.; Tuckerman, M. E.; Liu, J. Unified Efficient Thermostat Scheme for the Canonical Ensemble with Holonomic or Isokinetic Constraints via Molecular Dynamics. *J. Phys. Chem. A* **2019**, *123* (28), 6056–6079.
(14) Darden, T.; York, D.; Pedersen, L. Particle Mesh Ewald: An $N \cdot \log(N)$ Method for Ewald Sums in Large Systems. *The Journal of Chemical Physics* **1993**, *98* (12), 10089–10092.
(15) Smith, D. M. A.; Dupuis, M.; Vorpagel, E. R.; Straatsma, T. P. Characterization of Electronic Structure and Properties of a Bis(Histidine) Heme Model Complex. *J. Am. Chem. Soc.* **2003**, *125* (9), 2711–2717.
(16) Autenrieth, F.; Tajkhorshid, E.; Baudry, J.; Luthey-Schulten, Z. Classical Force Field Parameters for the Heme Prosthetic Group of Cytochrome c. *Journal of Computational Chemistry* **2004**, *25* (13), 1613–1622.
